# Regulators of Autophagosome Formation in *Drosophila* Muscles

**DOI:** 10.1371/journal.pgen.1005006

**Published:** 2015-02-18

**Authors:** Jonathan Zirin, Joppe Nieuwenhuis, Anastasia Samsonova, Rong Tao, Norbert Perrimon

**Affiliations:** 1 Department of Genetics, Harvard Medical School, Boston, Massachusetts, United States of America; 2 Howard Hughes Medical Institute, Harvard Medical School, Boston, Massachusetts, United States of America; University of Massachusetts Medical School, UNITED STATES

## Abstract

Given the diversity of autophagy targets and regulation, it is important to characterize autophagy in various cell types and conditions. We used a primary myocyte cell culture system to assay the role of putative autophagy regulators in the specific context of skeletal muscle. By treating the cultures with rapamycin (Rap) and chloroquine (CQ) we induced an autophagic response, fully suppressible by knockdown of core *ATG* genes. We screened *D. melanogaster* orthologs of a previously reported mammalian autophagy protein-protein interaction network, identifying several proteins required for autophagosome formation in muscle cells, including orthologs of the Rab regulators RabGap1 and Rab3Gap1. The screen also highlighted the critical roles of the proteasome and glycogen metabolism in regulating autophagy. Specifically, sustained proteasome inhibition inhibited autophagosome formation both in primary culture and larval skeletal muscle, even though autophagy normally acts to suppress ubiquitin aggregate formation in these tissues. In addition, analyses of glycogen metabolic genes in both primary cultured and larval muscles indicated that glycogen storage enhances the autophagic response to starvation, an important insight given the link between glycogen storage disorders, autophagy, and muscle function.

## Author Summary

Since the identification of the core autophagy genes in yeast, tissue culture cell lines have been the primary tool to evaluate the role and regulation of autophagy in higher organisms. However, since autophagy is a tissue-specific, context dependent process, stable cell lines can only give a limited view of the autophagic process. Here, we focus on the role of putative autophagy regulators in the specific context of the skeletal muscle, which has one of the most robust autophagy responses in mammals. We describe a fruitfly model of autophagy for skeletal muscles that integrates rapid genetic screening in primary cultured cells with robust *in vivo* validation in the larval muscle. We screened a set of genes previously linked to the autophagy pathway in humans, and identified both positive and negative regulators of autophagy. Our observation that genes involved in sugar metabolism impact muscle autophagy has important implications for both skeletal and cardiac myopathies associated with aberrant sugar storage. Surprisingly, we found that the proteasome is required to maintain autophagy in the muscle, suggesting that therapeutic treatments aiming to induce autophagy by proteasome inhibition must be carefully calibrated to ensure that the opposite phenotype does not occur.

## Introduction

Autophagy, literally “self-eating”, is a eukaryotic evolutionarily conserved mechanism for bulk degradation of organelles and long-lived proteins[1]. Upon induction, a portion of cytoplasm is enveloped in a closed double-membrane bound vesicle called the autophagosome, which subsequently fuses with the lysosome, where its inner membrane and contents are digested. The resulting degradation products are transported out of the lysosome and recycled for protein synthesis and ATP production. Studies in yeast have identified 35 *ATG* (autophagy-related) genes, many of which are conserved in higher organisms [1–3]. Induction occurs via the autophagy-related gene 1 (Atg1) complex, and membrane nucleation requires a complex containing Vps34 (the class III PI3K). Two different sets of ubiquitin-like protein conjugation systems, Atg8 and Atg5–Atg12 direct the expansion of the autophagosome around its target. Subsequently, fusion with the lysosome requires the endocytic Rab proteins, HOPS complex and SNARE machinery [2,4–9].

The Tor pathway, which links cellular nutritional status to metabolism and growth, regulates autophagy in response to starvation [10]. Tor represses the formation of autophagosomes by inhibition of Atg1, a function conserved from yeast to mammals [11]. In higher eukaryotes, the insulin/class I phosphoinositide 3-kinase (PI3K) pathway, upstream of Tor, links hormonal signaling to autophagy [12]. In addition, several other kinases have been shown to regulate autophagy either directly or via Tor, including cAMP-dependent protein kinase A (PKA), AMP-activated protein kinase (AMPK), calcium/calmodulin-dependent protein kinase 2, beta (CAMKK2/CaMKKβ) [12]. Despite the implication of these pathways in autophagy, there remain important questions about how upstream signaling events connect to the molecular machinery of autophagosome formation. Even the best characterized regulator, Tor, likely has additional substrates that regulate autophagy through unknown mechanisms [12,13]. The crosstalk between autophagy-regulating pathways is also poorly understood, as is the role of phosphatases and other regulatory proteins.

In addition to non-selective autophagy, which is primarily a starvation response, cells use selective autophagy for a variety of purposes, including remodeling to adapt to changing environmental/nutritional conditions, elimination of damaged organelles, and invading pathogens [14]. It has become clear in recent years that specific cargos are targeted to autophagosomes by receptor and adaptor proteins that connect the cargo to the core autophagy machinery [14]. Autophagy receptors have been identified in some cases of selective autophagy (e.g. viruses, bacteria, mitochondria, peroxisomes, midbody remnant), but remain elusive in other cases (e.g. lipid, ribosomes, endoplasmic reticulum) [14,15]. Even less is known about the molecules on the cargo that are recognized by the receptor proteins, although it is clear that ubiquitin can serve this purpose in some cases [14,15].

As an approach to identify new autophagy proteins that regulate autophagosome and substrate specificity, Behrends et al. used mass spectrometry to generate a network of autophagy protein-protein interactions [16]. Specifically, they expressed tagged versions of a subset of human proteins that were previously linked with autophagy or vesicle trafficking in HEK293T cells, and the coimmunoprecipitated proteins were identified by mass spectrometry. The resulting 409 high-confidence candidate interaction proteins were organized into an autophagy interaction network. While some of the identified interactors have been validated *in vivo*, the network as a whole has not been tested beyond the original transformed HEK293T and U2OS cell lines, and remains a relatively untapped resource.

Given the diversity of autophagy targets and regulation, it is important to characterize autophagy in various cell types and conditions. Thus, we decided to make use of a primary muscle cell culture system to assay the role of putative autophagy regulators in the specific context of this tissue. *Drosophila melanogaster* primary myoblast cultures can be easily prepared from gastrulating embryos in which myoblast cell fate is already defined. Myoblast fusion occurs in cultured cells, resulting in the formation of multi-nucleate myotubes [17]. These myotubes further differentiate to form a well-patterned myofibril structure according to the timeline of larval development [17]. Importantly, these myotubes actively contract in live culture, indicating that they are metabolically active and fully functional cells. Furthermore, these primary cultured muscles are suitable for RNAi screening, as simple bathing of the cells in dsRNA-containing medium is sufficient for an effective and specific RNAi effect [18]. Following the RNAi screen in primary culture cells, selected hits can then be further validated *in vivo*, as we have previously established the *D. melanogaster* larval muscle as a model system to study the role and regulation of autophagy in skeletal muscle [19,20].

To use primary muscle cells in an RNAi autophagy screen, we first developed a robust autophagy assay. By treating the cultures with rapamycin (Rap) and chloroquine (CQ) we were able to induce an autophagic response, fully suppressible by knockdown of core *ATG* genes. Rap binds to and inhibits Tor complex 1 (TORC1) [10], thus inducing autophagy by releasing the inhibitory effect of Tor on Atg1 and autophagosome nucleation [11], while CQ blocks flux, causing accumulation of autolysosomes and autophagosomes [20–22]. Next, we used this system to screen *D. melanogaster* orthologs of the mammalian autophagy network of Behrends et al. This approach allowed us to identify several genes required for autophagosome formation, including orthologs of the Rab effectors Rabgap1 and Rab3Gap. The screen also highlights the critical roles of the proteasome and glycogen metabolism in regulating autophagy in muscle. In particular, we found that sustained proteasome inhibition inhibits autophagosome formation, even though autophagy normally acts to suppress ubiquitin aggregate formation in the muscle. Our data suggests that this is due to a link between muscle activity, aggregate formation, and autophagy. Finally, our analyses of glycogen metabolic genes in both the primary cultured and larval muscles indicate that glycogen storage enhances the autophagic response to starvation, an important insight given the link between glycogen storage disorders, autophagy, and muscle function.

## Results

### An autophagy assay in cultured primary muscles

**Fig 1:**
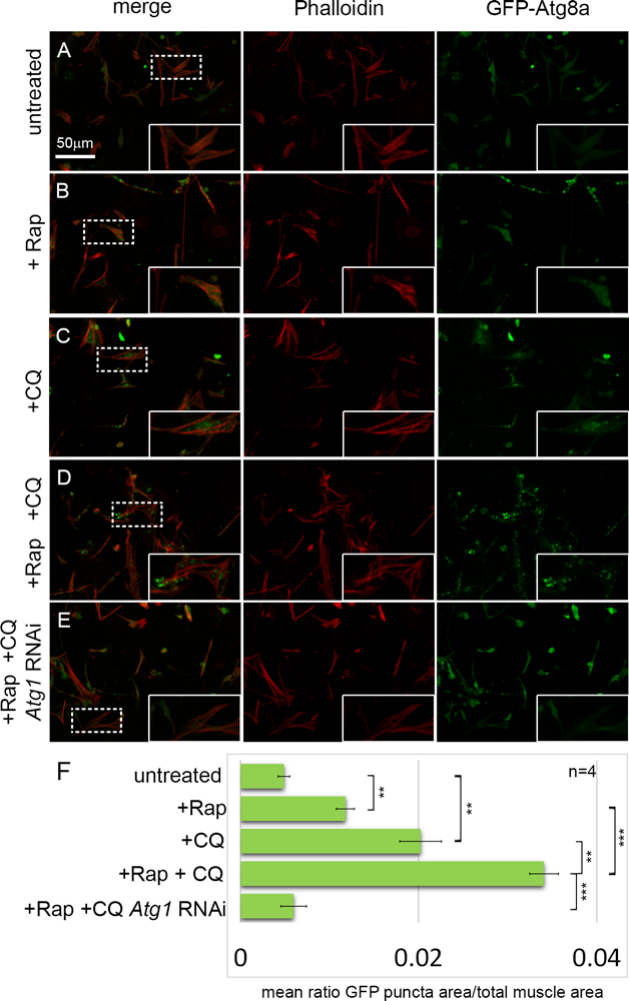
An assay for monitoring autophagy levels in primary cultured muscles. **(A-D)** Primary cultures from *D. melanogaster* embryos expressing GFP-Atg8a under the control of *Dmef2-Gal4*, stained with Phalloidin (actin) in red. Inset are magnifications of areas denoted by dotted line. **(A)** In untreated control cultures, GFP-Atg8a localizes throughout the muscle cytoplasm and nuclei. **(B)** Addition of rapamycin (Rap) for 12 hrs results in the formation of small GFP-Atg8a labeled punctae. **(C)** Addition of chloroquine (CQ) for 12 hrs results in the accumulation of large GFP-Atg8a labeled vesicles that localize around the nucleus and between the myofibers. **(D)** Addition of both CQ and Rap for 12hrs, triggers the formation of greater numbers of enlarged vesicles in the muscles than either drug alone. **(E)** Knockdown of the core autophagy gene, *Atg1*, prevents the formation of GFP-Atg8a vesicles in CQ+Rap treated cultures. **(F)** Quantification of the mean ratio of GFP-Atg8a punctae area to total muscle area per well (n = 4). Error bars indicate SEM with **p<.01; ***p<.001.

To establish an autophagy assay in cultured primary muscles, we established cultures from *Dmef2-Gal4*, *UAS-GFP-Atg8a* embryos (Fig. 1). As previously described, these cultures contain a mixed population of cells, including neurons, fat, gut, and muscle cells [23,24]. Use of the *Dmef2-Gal4* driver ensures that GFP-Atg8a fusion protein is expressed only in muscle cells, allowing us to analyze autophagy specifically in this one cell type. Cultures were also stained with Phalloidin (F-actin) to detect the striated sarcomeric structures. In untreated control cultures, GFP-Atg8a was distributed throughout the cytoplasm of muscle cells as well as nuclei (Fig. 1A, F). The presence of GFP-Atg8a in the nucleus is consistent with previous reports of nuclear localization of both GFP-tagged and endogenous LC3 in mammals [25–27]. To induce autophagy, we treated the cultures with rapamycin (Rap), a Tor inhibitor, which has been successfully used to activate autophagy in both *D. melanogaster* and higher organisms [10,28]. After 12 hrs of Rap treatment (200 nM), we observed the formation of GFP-Atg8a punctae in the muscles, consistent with the drug inducing autophagy (Fig. 1B, F). However, the small size of the punctae, and the poor contrast between the putative autophagosomes and the GFP-Atg8a background staining, indicated that this phenotype would not be suitable for screening. Thus, to generate a more robust phenotype, we treated the cultures with chloroquine (CQ). CQ and its closely related analog hydroxychloroquine are 4-aminoquinoline compounds widely used to treat malaria, rheumatoid arthritis and lupus erythematosus [29–31]. The drugs are highly lysosomotropic, causing an increase in lysosomal pH, blocking autophagic flux, leading to accumulation of autophagosomes and autolysosomes [21,22]. We previously showed that treatment of *D. melanogaster* larvae with CQ in conjunction with starvation was able to induce a robust autophagy phenotype in skeletal muscles *in vivo* [19,20]. In cultured cells, 12 hrs of CQ treatment (200 μM) induced the formation of large GFP-Atg8a punctae (Fig. 1C, F). The induction of autophagy by CQ treatment is consistent with the view that CQ inhibits mTORC1 activity in mammalian cells [32]. Addition of both CQ and Rap together increased GFP-Atg8a punctae area more than either drug alone (Fig. 1D, F), indicating that CQ was able to inhibit the autophagic flux induced by Rap. The resulting enlarged punctae were distributed throughout the cytoplasm, generally in between the myofibers marked by Phalloidin stain. Next, we tested whether the formation of the GFP-Atg8a punctae observed in the cultured muscles requires a functional autophagy pathway. This is especially important as both endogenous Atg8 and GFP-Atg8a fusion proteins tend to be incorporated into intracellular protein aggregates, independent of autophagy, and therefore Atg8 or GFP-Atg8a positive punctae could represent either an aggregate or a bone fide autophagosome. Consistent with the latter interpretation, knockdown of the core autophagy gene *Atg1* was able to completely suppress the formation of GFP-Atg8a punctae in the cultured Rap+CQ treated muscles (Fig. 1E, F).

### An RNAi screen for autophagy genes

**Fig 2:**
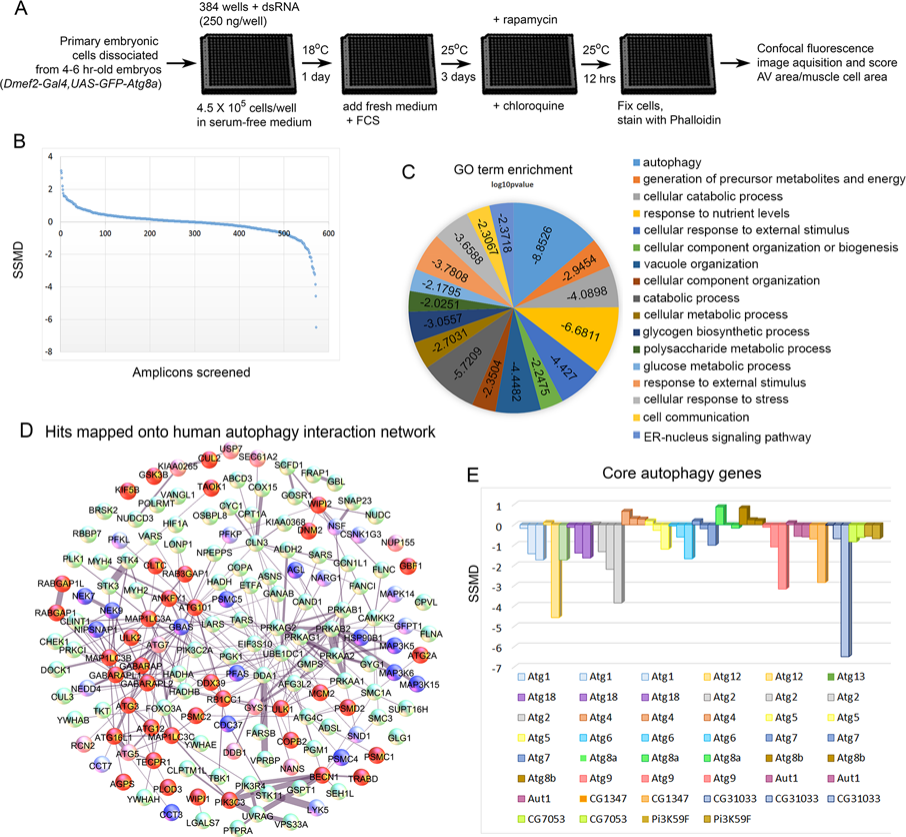
Screen of *D. melanogaster* autophagosome network orthologs identifies genes essential for autophagy in skeletal muscles. **A)** Outline of the RNAi screen in primary cultured muscles. **B)** Results of RNAi knockdown screen on autophagic vesicle area/primary muscle cell area. Y-axis is the strictly standardized mean difference (SSMD) scores for each amplicon tested (X-axis). **C)** GO term enrichment (biological process) of human orthologs hits. Scores on pie chart indicate the log10 p-value of enrichment. **D)** Hits mapped onto the 187 human genes used as the basis for screening in *D. melanogaster*. Protein-protein interactions were identified from the set of 409 proteins identified in mammalian cells[16]. For gene knockdowns that inhibited autophagy, dark red indicates that two amplicons had a score < -0.5, while light red indicates that only one amplicon had a significant SSMD score, but that it was < -1. For gene knockdowns that increased autophagy, dark blue indicates that two amplicons had an SSMD score > 0.5, while light blue indicates that only one amplicon had a significant SSMD score, but that it was > 1. **(E)** At least one amplicon scored as a hit for each of the core autophagy genes tested, with the exception of *Atg8*, which is our marker for autophagy and is being overexpressed with *Dmef2-Gal4*, and *Atg4*.

Having established a robust autophagic response in primary cultured muscles and shown by RNAi that it requires components of the autophagy pathway, we assembled a library of dsRNAs genes targeting the *D. melanogaster* orthologs of a mammalian autophagy network [16]. The complete list of mammalian genes and their *D. melanogaster* orthologs included in our set is shown in S1 Table. For the screen-outlined in Fig. 2A, genes were targeted by multiple independent dsRNAs and at least three replicates of each 384 well plate were screened. We scored autophagy levels by calculating the GFP-Atg8a vesicle area per muscle cell area for a given well (S1 Fig.), and analyzed the strength of the phenotype using the Strictly Standardized Mean Difference (SSMD) method [33,34]. Developed for analysis of RNAi screen data sets, SSMD is the ratio between the difference of the means and the standard deviation of the difference between positive and negative controls (see Materials and Methods for details). Using this method, negative control *LacZ* RNAi consistently failed to score, while positive control *Atg18* RNAi consistently gave a score <-1, indicating strong autophagy suppression (S2 Fig.). A plot of the SSMD scores for each amplicon shows that we identified dsRNAs that caused both suppression and enhancement of autophagosome formation (Fig. 2B). We identified 47 genes that when knocked down, suppressed autophagosome formation, and 25 genes with the opposite phenotype (Table 1, Table 2, S1 Table). It is important to note that in using CQ to boost the autophagy signal we likely masked the effect of some genes that normally promote autophagosome-lysosome fusion and subsequent flux of autolysosomes. Further, by treating with Rap we will also masked genes involved in the regulation of autophagy upstream of the Tor pathway. Nonetheless, of 17 core autophagy genes included in the set only *Atg8a* (which we are overexpressing in the GFP-tagged form) and *Atg4* failed to score (Fig. 2D-E), indicating the suitability of our approach for identifying genes required for autophagosome formation. Overall, at least 40% of the genes included in the screen are good candidates for autophagy pathway components/regulators in the *D. melanogaster* muscle. Fig. 2D shows a projection of the hits that score in our assay onto the 187 human genes from the network of Behrends et al. (2010). Gene Ontology (GO) analysis identified autophagy and cellular catabolism as the two most enriched terms (Fig. 2C), which is expected considering that our starting set was derived from an autophagy protein-protein interaction network. Interestingly, glucose and polysaccharide metabolism and glycogen biosynthesis were also enriched terms, suggesting that sugar metabolism plays an important role in muscle autophagy.

**Table 1:** List of genes with two or more dsRNAs scoring in primary culture screen.

*D. melanogaster* genes	*H. sapiens* genes	Direction of autophagy regulation
*Atg1*	*ULK1/ULK2*	positive
*Atg18*	*WIPI1/WIPI2*	positive
*Atg2*	*ATG2A/ATG2B*	positive
*Atg6*	*BECN1*	positive
*Atg9*	*ATG9A/B*	positive
*Aut1*	*ATG3*	positive
*CG10253*	*AGPS*	positive
*CG12360*	*TRABD*	positive
*CG1347*	*RB1CC1*	positive
*CG31033*	*ATG16L1*	positive
*CG31935*	*RAB3GAP1*	positive
*CG32226*	*TECPR1*	positive
*CG41099*	*ANKFY1*	positive
*CG6199*	*PLOD3*	positive
*CG7053*	*ATG101*	positive
*CG7112*	*RABGAP1/1L*	positive
*CG8678*	*WIPI1/WIPI2*	positive
*Chc*	*CLTC*	positive
*Cul-2*	*CUL2*	positive
*garz*	*GBF1*	positive
*Hel25E*	*DDX39B*	positive
*Khc*	*KIF5B*	positive
*Mcm2*	*MCM2*	positive
*Pi3K59F*	*PIK3C3*	positive
*Pros26.4*	*PSMC1*	positive
*Rab3-GAP*	*RAB3GAP2*	positive
*Rpn1*	*PSMD2*	positive
*Rpt1*	*PSMC2*	positive
*sgg*	*GSK3B*	positive
*shi*	*DNM2*	positive
*Tao-1*	*TAOK1*	positive
*ade2*	*PFAS*	negative
*Cctgamma*	*CCT3*	negative
*Cdc37*	*CDC37*	negative
*CG42233*	*WDR22*	negative
*CG9485*	*AGL*	negative
*Gp93*	*HSP90B1*	negative
*Nek2*	*NEK7*	negative
*Nipsnap*	*GBAS/NIPSNAP1*	negative
*pk*	*PRICKLE2*	negative
*Pk92B*	*MAP3K6/MAP3K15/MAP3K5*	negative
*Pros45*	*PSMC5*	negative
*Rpt3R*	*PSMC4*	negative

*D. melanogaster* genes (column A) are matched to their human orthologs (column B). Positive autophagy regulators are genes whose knockdown caused reduced autophagosome formation (SSMD<-0.5). Negative autophagy regulators are genes whose knockdown caused increased autophagosome formation (SSMD>0.5). Only genes with two or more dsRNAs meeting the cutoff are included in the table.

**Table 2:** List of genes with one dsRNA scoring in primary culture screen.

*D. melanogaster* genes	*H. sapiens* genes	Direction of autophagy regulation
*Atg12*	*ATG12*	positive
*Atg13*	*ATG13*	positive
*Atg5*	*ATG5*	positive
*Atg7*	*ATG7*	positive
*CG13745*	*FANCI*	positive
*CG6904*	*GYS1*	positive
*gish*	*CSNK1G3*	positive
*Mhc*	*MYH2*	positive
*Nup154*	*NUP155*	positive
*pic*	*DDB1*	positive
*Rab1*	*RAB1A*	positive
*Sas*	*NANS*	positive
*Sec61alpha*	*SEC61A2*	positive
*slim*	*KIAA0265*	positive
*Usp7*	*USP7*	positive
*Vps16A*	*PTPRA*	positive
*aPKC*	*PRKCI*	negative
*CG3590*	*ADSL*	negative
*CG9784*	*INPP5K*	negative
*Gfat1*	*GFPT1*	negative
*Mpk2*	*MAPK14*	negative
*Nat1*	*NARG1*	negative
*Nedd4*	*NEDD4*	negative
*Nsf2*	*NSF*	negative
*Pfk*	*PFKL/PFKP*	negative
*Rpt6R*	*PSMC5*	negative
*Stlk*	*LYK5*	negative
*Tcp-1eta*	*CCT7*	negative
*Tudor-SN*	*SND1*	negative

*D. melanogaster* genes (column A) are matched to their human orthologs (column B). Positive autophagy regulators are genes whose knockdown caused reduced autophagosome formation (SSMD<-1). Negative autophagy regulators are genes whose knockdown caused increased autophagosome formation (SSMD>1). Only genes with one dsRNA meeting the cutoff are included in the table. Genes are included in the table if a single dsRNA met the threshold (±1.0 SSMD) and no other dsRNA targeting that gene met the threshold (±0.5 SSMD) in the opposite direction.

### Extended proteasome knockdown blocks the autophagy induction normally seen with short-term proteasome inhibition

**Fig 3:**
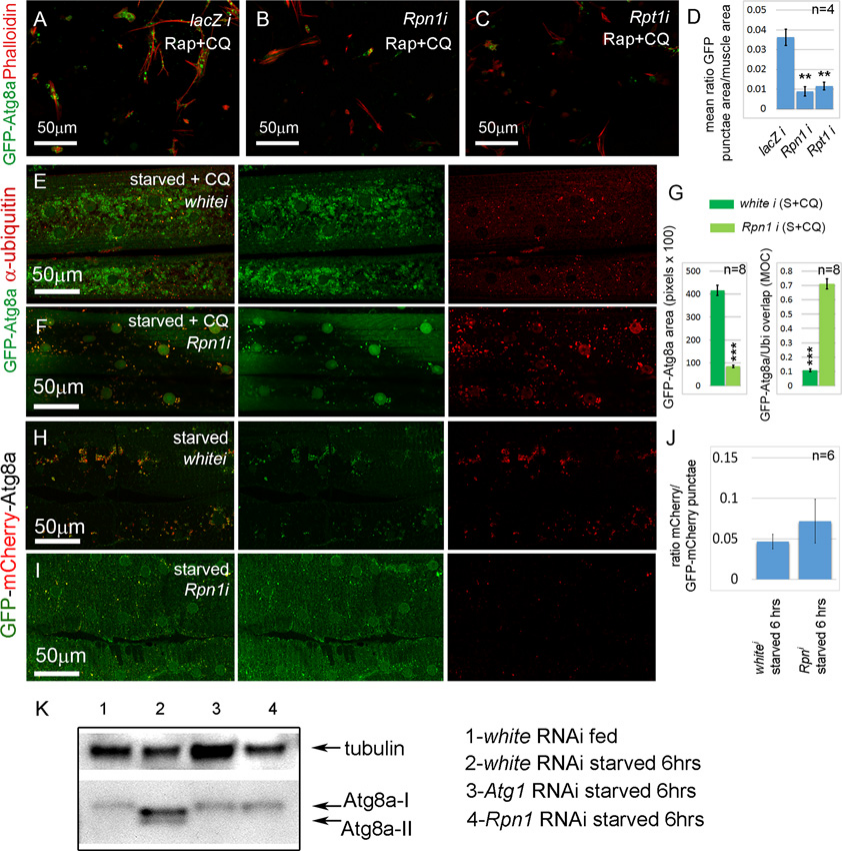
Sustained proteasome dysfunction inhibits autophagosome formation. **(A)** Primary cultured muscles with control *lacZ* RNAi, treated for 12 hrs with Rap+CQ. Note the formation of large GFP-Atg8a vesicles throughout the muscles. GFP-Atg8a vesicles are much reduced in primary cultured muscles with *Rpn1 (PSMD2)* RNAi **(B)** or *Rpt1 (PSMC2)* RNAi **(C)**, treated for 12 hrs with Rap+CQ. **(D)** Quantification for A-C of the mean ratio of GFP-Atg8a punctae area to total muscle area per well (n = 4). SEM is indicated with **p<.01. **(E)** Control *white* RNAi muscles starved and treated with CQ accumulate GFP-Atg8a punctae, but very few colocalize with ubiquitin (Ubi). **(F)** Knockdown of *Rpn1* in starved + CQ muscle strongly inhibits the formation of GFP-Atg8a punctae. The remainder colocalize with ubiquitin. **(G)** Quantification of the mean GFP-Atg8a punctae area and Mander’s overlap coefficient for GFP-Atg8a and Ubi for genotypes shown in E and F. Error bars indicate SEM for n = 8 ventral longitudinal muscles from individual animals, with p-values for *Rpn1* RNAi relative to *white* RNAi control (***p<.001). **(H, I)** Autophagosome number is reduced, but autophagic flux is not significantly altered in starved *Rpn1* vs. *white* RNAi muscles expressing GFP-mCherry-Atg8. **(J)** Flux measurement is shown on the right as the ratio of mCherry punctae to GFP-mCherry (n = 6). SEM is indicated. **(K)** Anti-Atg8a Western blot of third instar muscle lysate from *Dmef2-Gal4; UAS-RNAi animals*. In fed *white* RNAi control, endogenous Atg8a appears as a single band (Atg8a-I). 6 hrs starvation induces lipid modification of Atg8a and the appearance of a second band (Atg8a-II). Knockdown of *Atg1* or *Rpn1* inhibits Atg8a modification, indicating reduced autophagy levels. In all panels green = GFP-Atg8a. Red = Phalloidin (A-C), anti-ubiquitin (E, F), or mCherry (G, H).

Interestingly, among the positive regulator candidates of autophagy were the *D. melanogaster* orthologs of the human proteasome components *PSMC2* (*D.m*. gene *Rpt1*), and *PSMD2* (*D.m*. gene *Rpn1*). It has been widely reported that proteasome inhibition causes increased autophagy [35], but dsRNAs targeting these core components of the proteasome complex inhibited rather than induced the formation of GFP-Atg8a punctae in the primary cultured muscles (Table 1 and Fig. 3A-D). To determine whether this also occurred *in vivo* we examined the effect of proteasome knockdown in the larval skeletal musculature of *Dmef2-Gal4*, *UAS-GFP-Atg8a* animals. In fed animals, GFP-Atg8a was distributed throughout the muscle cytoplasm and nuclei (S3A Fig.). In contrast, in larvae starved on low nutrient food for 6 hrs, GFP-Atg8a localized to small punctae surrounding the nuclei and between the myofibrils (S3B Fig.). Starvation on low nutrient food + CQ for 6 hrs caused induction of much larger and more numerous punctae than those in the non-treated muscle, but were similarly distributed around the nucleus and between myofibrils (S3C Fig.). Next, we compared the effect of *Rpn1* RNAi to control *white* RNAi. As expected, the control muscles accumulated large GFP-Atg8a punctae when starved and treated with CQ (Fig. 3E, G). Staining with an antibody that recognizes mono and poly-ubiquitinated proteins revealed that only a small proportion of the GFP-Atg8a colocalized with ubiquitin (Fig. 3E, G). In contrast, *Rpn1* knockdown reduced the number of GFP-Atg8a punctae, and dramatically increased the proportion that colocalized with ubiquitin (Fig. 3F, G). To determine whether this finding was unique to *Rpn1*, we tested by RNAi the effect of knocking down other proteasome subunits. Consistent with the *Rpn1* result, several components of both the 19 and 20S proteasome subunits gave a similar phenotype (S4 Fig.). Thus, sustained knockdown of the proteasome both in cultured primary muscles and in the larval musculature inhibits autophagy.

We next assayed the effect of proteasome inhibition in the absence of CQ on autophagosome and autolysosome formation and flux. Using *Dmef2-Gal4* we drove expression of *a UAS-GFP-mCherry-Atg8a* transgene [36]. The double-tagged Atg8a protein fluoresces yellow in autophagosomes, while the GFP is quenched in the acidic environment of the autolysosomes, leaving these structures red. In fed animals, GFP and mCherry colocalized throughout the cytoplasm and nuclei of the muscle. (S3F Fig.). Starvation for 6hrs induced punctae labeled by both GFP and mCherry (S3G, J Fig.), indicating that acidification of vesicles had not yet occurred. The proportion of vesicles labeled only by mCherry increased after 8 hrs starvation (S3H, J Fig.), and by 10 hrs after starvation most punctae lacked GFP fluorescence (S3I, J Fig.). Using this system we compared the effect of *Rpn1* RNAi to *white* RNAi control muscles. As expected, knockdown of *white* had no effect on the ratio of the mCherry and GFP signals after 6hrs of starvation (Fig. 3H, J, S3L Fig.). *Rpn1* knockdown significantly reduced the number of both GFP and mCherry labeled vesicles, but did not affect their ratio (Fig. 3I, J, S3K, S3L Fig.), indicating that autophagic flux was not altered. Consistent with these results, we also found that *Rpn1* knockdown reduced the number of GFP-Lamp1 labeled vesicles in both starved and starved +CQ larval muscles (S5 Fig.).

To eliminate the possibility that the above phenotypes are an artifact of overexpressed *Atg8a* transgenes, we next performed a Western blot on larval muscle lysate using a previously described Atg8a antibody [37] (Fig. 3K). In fed *Dmef2-Gal4/UAS-white RNAi* larvae, endogenous Atg8a appeared as a single band (Atg8a-I). Starvation of *Dmef2-Gal4/UAS-white RNAi* larvae for 6 hrs on low nutrient food induced the appearance of an additional smaller band (Atg8a-II), which represents lipidated Atg8a and indicates increased autophagy [38]. We also observed a slight change in the mobility of the Atg8a-I band in the starved muscle relative to fed muscle. No Atg8a-II band appeared in either starved *Dmef2-Gal4/UAS-Atg1 RNAi* larvae or starved *Dmef2-Gal4/UAS-Rpn1 RNAi* larvae, indicating reduced autophagy, consistent with the GFP-Atg8a immunofluorescence phenotypes in both primary cultured muscles and larval muscles.

**Fig 4:**
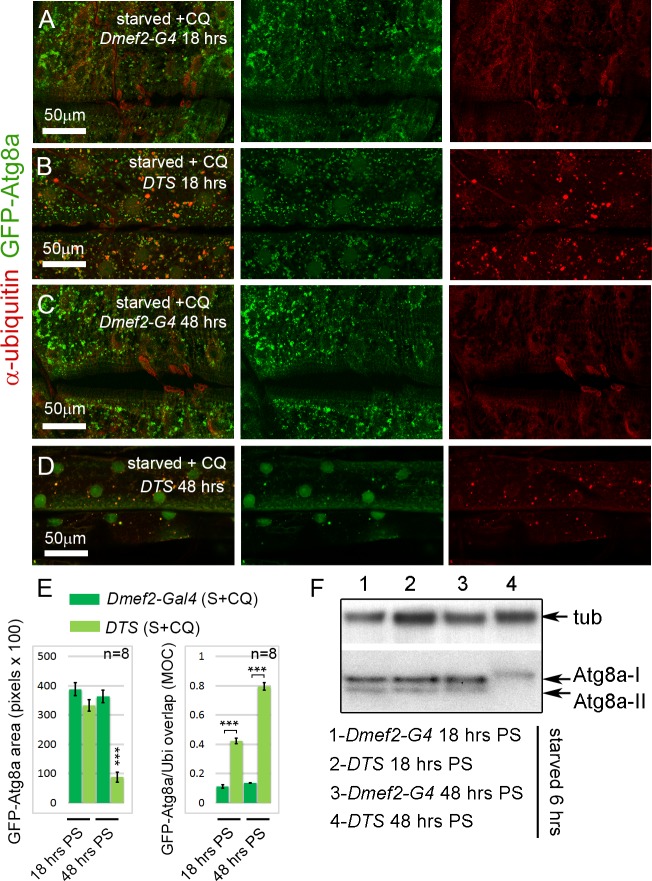
Short-term, but not long-term, proteasome disruption triggers autophagic clearance of aggregates **(A-D)** Inhibition of the proteasome by overexpression of a temperature sensitive proteasome mutant (DTS). *Dmef2-Gal4*, *UAS-GFP-Atg8a* control and *UAS-DTS; Dmef2-Gal4*, *UAS-GFP-Atg8a* and animals were shifted to the restrictive temperature then starved and CQ treated 6 hrs prior to dissection. 18 hrs post-shift (PS), both control **(A)** and DTS muscles **(B)** accumulate GFP-Atg8a punctae, but the latter also accumulate ubiquitin (Ubi) aggregates. 48 hrs PS, control muscles **(C)** maintain strong autophagy induction, but DTS mutant muscles **(D)** have reduced GFP-Atg8a punctae accumulation, similar to the *Rpn1* knockdown phenotype. **(E)** Quantification of the mean GFP-Atg8a punctae and Mander’s overlap coefficient for GFP-Atg8a and Ubi for genotypes shown in A-D (SEM is indicated for n = 8 ventral longitudinal muscles from individual animals with p-values of DTS relative to control). Dark green = *Dmef2-Gal4*, *UAS-GFP-Atg8a*; Light green = *UAS-DTS; Dmef2-Gal4*, *UAS-GFP-Atg8a*. **(F)** Western blot analysis of endogenous Atg8a from third instar muscle lysate. 6 hrs starvation induces lipidated Atg8a-II in *Dmef2-Gal4* control at both 18 hrs and 48 hrs PS, but not in *DTS* at 48 hrs PS.

Increased autophagy has been previously reported in other models of proteasome inhibition. Rap-induced autophagy in either cell culture or mice has been shown to protect against cell death caused by proteasome inhibition [39] and upregulation of autophagy has been shown to protect against loss of proteasome activity in *D. melanogaster* [40]. Thus, we hypothesized that the extended time period of proteasome inhibition might explain the difference between these previous studies and the reduced autophagy observed with proteasome knockdown. To test this hypothesis, we used a dominant temperature sensitive allele of the *Pros26* gene (*DTS5*), the *D. melanogaster* ortholog of the human *PSMB1* gene, encoding the proteasome b6 subunit [41,42]. Overexpression of *UAS-DTS5* allowed us to modulate the time period of proteasome disruption, since the allele only inhibits the proteasome at the restrictive temperature. When shifted to the restrictive temperature for 18 hrs, *UAS-DTS5; Dmef2-Gal4*, *UAS-GFP-Atg8a* larvae, starved and treated with CQ 6hrs prior to dissection, accumulated large numbers of GFP-Atg8a punctae in their muscles similar to control *Dmef2-Gal4*, *UAS-GFP-Atg8a* larvae (Fig. 4A, B, E). Compared to the control larvae, however, we observed a significant increase in colocalization between GFP-Atg8a and ubiquitin in *UAS-DTS5; Dmef2-Gal4*, *UAS-GFP-Atg8a* larvae (Fig. 4A, B, E). When we performed the same experiment with a longer 48 hrs temperature shift, GFP-Atg8a punctae formation was dramatically reduced in DTS compared to control muscles, resembling the phenotype associated with *Rpn1* knockdown (Fig. 3F, 4C-E). Surprisingly given the extended period of proteasome inhibition, the amount of ubiquitin aggregates appear reduced compared to the shorter 18 hrs temperature shift (Fig. 4B, D). We confirmed the effect of the DTS mutant on endogenous Atg8a by Western blot (Fig. 4F). Following 6 hrs of starvation lipidated Atg8a was observed in *Dmef2-Gal4* control muscles at both 18 and 48 hrs PS. *UAS-DTS5; Dmef2-Gal4* muscles also showed normal autophagy induction at 18 hrs PS, but at 48 hrs PS only the non-lipidated Atg8a-I form was present.

**Fig 5:**
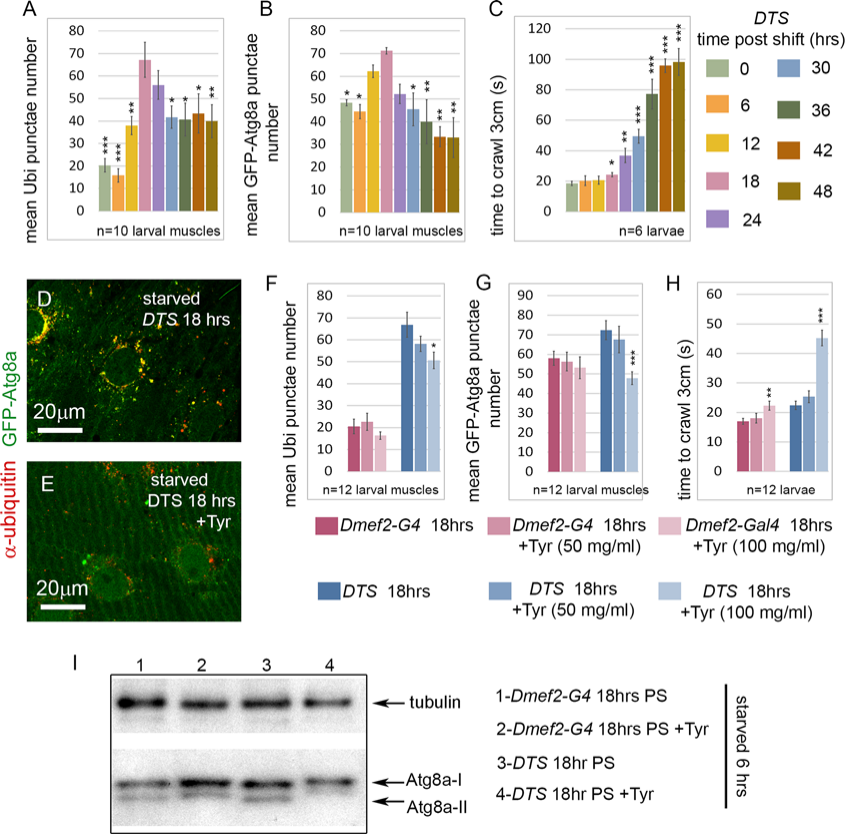
Tyramine (Tyr) treatment reduces autophagy induced by proteasome inhibition. **(A-C)** Time course of effect of temperature shift on aggregates, autophagosomes, and larval locomotion in *UAS-DTS; Dmef2-Gal4*, *UAS-GFP-Atg8a* animals starved 6 hrs prior to analysis. **(A)** The number of ubiquitin aggregates peaks at 18–24 hrs PS, and decreases significantly from 18 to 48 hrs PS (n = 10 and p-values shown relative to 18 hrs PS). **(B)** The number of GFP-Atg8a punctae peaks at 12–24 hrs PS and decreases significantly from 18 to 48 hrs PS (n = 10 and p-values shown relative to 18 hrs PS). **(C)** Larval locomotion, measured as the time to crawl 3cm, gradually and significantly worsens from 18 to 48 hrs PS (n = 6 and p-values shown relative to 0 hrs PS. **(D-E)** tyramine (Tyr) treatment reduces GFP-Atg8a punctae in larval muscle. *UAS-DTS; Dmef2-Gal4*, *UAS-GFP-Atg8a* animals were placed on 100mg/ml Tyr **(D)** or control food **(E),** shifted to restrictive temperature for 18hrs, and starved 6 hrs prior to dissection. **(F-H)** Effect of Tyr treatment on aggregates, autophagosomes, and larval locomotion in *UAS-DTS; Dmef2-Gal4*, *UAS-GFP-Atg8a* and *Dmef2-Gal4*, *UAS-GFP-Atg8a* animals, shifted to restrictive temperature for 18hrs, and starved 6 hrs prior to dissection. Aggregate **(F)** and GFP-Atg8a punctae **(G)** numbers in muscles are significantly reduced with 100 mg/ml Tyr treatment in DTS but not control animals 18 hrs post-shift (PS). **(H)** 100 mg/ml Tyr treatment significantly worsens larval locomotion in both control and DTS animals 18 hrs PS. n = 12 and p-values shown relative to non-Tyr treated animals. For all measurements, SEM is shown with *p<.05; **p<.01; ***p<.001. **(I)** Western blot analysis of endogenous Atg8a from third instar muscle lysate 18 hrs PS. 6 hrs starvation induces lipidated Atg8a-II in *Dmef2-G4* control with or without 100 mg/ml Tyr. After 6hrs starvation, Atg8a-II levels are reduced in *DTS* + 100 mg/ml Tyr relative to untreated *DTS*.

The finding that proteasome inhibition did not simply cause progressive increase of aggregates in the muscle led us to perform a time course analysis of aggregate number, GFP-Atg8a punctae number, and larval locomotion from 0 to 48 hrs after shifting *UAS-DTS5; Dmef2-Gal4*, *UAS-GFP-Atg8a* larvae to the restrictive temperature. Prior to dissection, each animal was starved for 6 hrs to heighten autophagy induction. Both the number of ubiquitin aggregates and autophagosomes peaked between 12–24 hrs post shift (PS), then decreased significantly from 24 to 48 hrs PS (Fig. 5A, B). Larval crawling time, gradually and significantly worsened from 18 to 48 hrs PS, indicating that proteasome inhibition causes a strong locomotion defect (Fig. 5C). Given these results, we hypothesized that reduced muscle activity/locomotion might influence aggregate formation and autophagy induction in response to proteasome inhibition. To test this, we fed both *Dmef2-Gal4*, *UAS-GFP-Atg8a and UAS-DTS5;Dmef2-Gal4*, *UAS-GFP-Atg8a* animals with the biogenic amine tyramine (Tyr), which has previously been shown to cause reduced locomotion in *D. melanogaster* larvae [43] (Fig. 5D-H). At the restrictive temperature this caused a significant increase in crawling time of the *Dmef2-Gal4*, *UAS-GFP-Atg8a* larvae and doubled the crawling time of the *UAS-DTS5; Dmef2-Gal4*, *UAS-GFP-Atg8a* larvae (Fig. 5H). Consistent with our hypothesis, Tyr treatment significantly reduced autophagosome number and aggregate number 18 hrs PS in the DTS mutant larvae (Fig. 5E-G). No significant change was observed for the *Dmef2-Gal4*, *UAS-GFP-Atg8a* control (Fig. 5D, F, G). We confirmed these results by Western blot of endogenous Atg8a (Fig. 5I). Tyr treatment reduced starvation-induced Atg8a-II formation in *UAS-DTS5; Dmef2-Gal4* but not control *Dmef2-Gal4* muscles 18 hrs PS. Taken together, these results indicate that autophagy of protein aggregates is triggered by proteasome inhibition, but reduced larval locomotion attenuates this response if inhibition is prolonged.

### The *D. melanogaster* orthologs of RABGAP1, RAB3GAP1, and the Rab effector protein ANKFY1 are required in vivo for autophagy in the muscle

**Fig 6:**
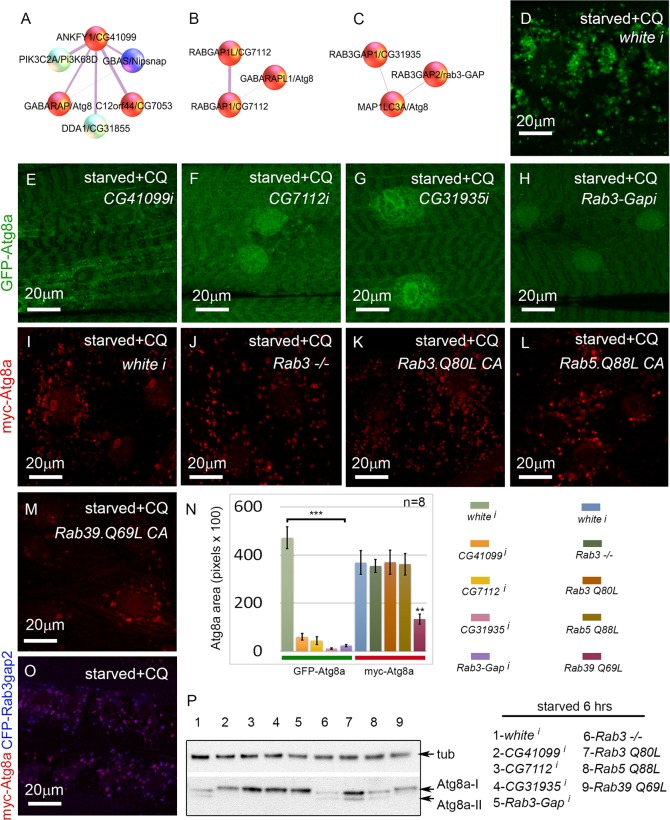
Rab GTPase-activating proteins and a Rab effector protein are required for autophagy *in vivo*. **(A-C)** Protein interactors of ANKFY1 (*D.m*. gene = *CG41099*), RABGAP1 and RABGAP1L (*D.m* gene = *CG7112*), RAB3GAP1 (*D.m*. gene = *CG31935*), and RAB3GAP2 (*D.m*. gene = *Rab3-GAP*), identified in mammalian mass spec. Note that each of these proteins interacts with an Atg8a ortholog. *D. melanogaster* orthologs of other protein interactors of ANKYF1 (*D.m*. genes *Nipsnap* and *CG7053*) were also identified as hits in the primary muscle screen. Based on our RNAi experiments red spheres are assigned to genes/proteins that promote autophagosome formation and/or expansion; blue spheres are assigned to genes/proteins that suppress autophagosome formation and/or expansion. **(D-H)** *Dmef2-Gal4* drives expression of *UAS-GFP-Atg8a* in animals starved on low nutrient food for 6 hrs + 2.5 mg/ml CQ for 6 hrs. RNAi knockdown of *CG41099* **(E)**, *CG7112* **(F)**, *CG31935* **(G)**, and *Rab3-GAP* **(H)**, blocks the formation of GFP-Atg8a labeled autophagosomes compared to knockdown of *white* (D). **(I-M)** *Dmef2-Gal4* drives expression of *UAS-myc-Atg8a* in animals starved on low nutrient food for 6 hrs + 2.5 mg/ml CQ for 6 hrs. The effect of the RAB3GAPs is not via Rab3 or Rab5 function, as a *Rab3* null mutation **(J)**, expression of a constitutive active form of Rab3 (Q80L) **(K)** or Rab5 (Q88L) **(L)** has no effect on muscle autophagy compared to control **(I)**. Expression of a constitutive active form of Rab39 (Q69L) **(M)** reduces myc-Atg8a labeled vesicles. **(N)** Quantification of autophagy phenotypes from panels D-M. SEM is indicated, with n = 8 ventral longitudinal muscles from individual animals and **p<.01; ***p<.001. **(O)** Atg8a and Rab3-GAP colocalize to autophagosomes. *Dmef2-Gal4* drives expression of *UAS-myc-Atg8a* and *UAS-CFP-Rab3-GAP* in animals starved and treated with CQ for 6 hrs. In all panels green = GFP-Atg8a and red = myc-Atg8a, and blue = CFP-Rab3Gap2. **(P)** Anti-Atg8a Western blot of third instar muscle lysate from *Dmef2-Gal4* driving the RNAi or overexpression constructs shown in D-N. Total levels of endogenous Atg8a are variable, but only knockdown of *CG41099*, *CG7112*, *CG31935*, *Rab3-GAP*, or overexpression of *Rab39 (Q69L)* block induction of lipidated Atg8a-II following 6 hrs starvation.

In addition to proteasome components, another well-represented category of screen hits were involved in vesicle trafficking. Many of these could be included in the core autophagy pathway, but others have been uncharacterized in the context of autophagy. This latter group includes the Rab GTPase-activating proteins *CG7112 (H. sapiens RABGAP1* and *RABGAP1L*), *CG31935 (H. sapiens RAB3GAP1)*, and *Rab3-GAP (H. sapiens RAB3GAP2)*, and the Rab effector *CG41099 (H. sapiens ANKFY1)* (Fig. 6A-C). ANKFY1 has been previously identified as a Rab5 effector that localizes to early endosomes and stimulates their fusion activity [44]. In the primary culture screen, knockdown of *CG41099* inhibited autophagosome formation (Table 1). Consistent with this data, knockdown of *CG41099* in larval muscles strongly inhibited the formation of enlarged GFP-Atg8a vesicles induced by starvation and CQ treatment (Fig. 6E, N). Interestingly, this phenotype appears to be due to a change in autophagic flux, as *CG41099* knockdown caused the ratio of mCherry to GFP fluorescence to significantly increase when assayed with the GFP-mCherry-Atg8a reporter (S3L Fig.). We likewise tested the *in vivo* function of *CG7112*, *CG31935* and *Rab3-GAP*, each of which identified as a positive regulator of autophagy in the primary culture screen. Knockdown of any of these genes strongly inhibited starvation induced autophagosome formation in the larval muscle with or without CQ treatment (Fig. 6F-H, N, S3K Fig.) and had no significant effect on autophagic flux (S3L Fig.).

Interestingly, *D. melanogaster* CG31935 and Rab3-GAP do not appear to influence autophagy via regulation of Rab3 activity, as larvae expressing a constitutive active form of Rab3 showed no change in the accumulation of myc-Atg8a-labeled autophagosomes (Fig. 6I, K, N). Larvae homozygous for a null allele of *Rab3* had a statistically insignificant inhibition of autophagosome formation compared to control larvae (Fig. 6J, N). We also tested the constitutive active forms of Rab5 and Rab39, as orthologs of these have been shown to interact with mammalian RAB3GAP1 in vitro [45]. Larvae expressing Rab5-CA had normal levels of autophagy in starved +CQ muscles (Fig. 6L, N). Interestingly, Rab39-CA overexpression significantly reduced autophagy similar to *CG31935* and *Rab3-GAP* RNAi (Fig. 6M-N). We confirmed the autophagy phenotypes of the above Rab, Rab-GAP, and Rab effector genes by Atg8a Western blot (Fig. 6P). Consistent with the immunofluorescence data, knockdown of *CG41099*, *CG7112*, *CG31935*, *Rab3-Gap* and overexpression of *Rab39-CA* reduced Atg8a lipidation in response to 6 hrs starvation. The human protein-protein interaction data suggests that Rab3-GAP might also interact with Atg8a (Fig. 6C). Consistent with this, we found nearly perfect colocalization in the larval muscles between overexpressed myc-Atg8a and CFP-Rab3-GAP in punctae induced by starvation and CQ treatment (Fig. 6O). Taken together, these results show that Rab GTPase-activating proteins, Rab39, and a Rab effector protein are required for autophagy *in vivo*.

### Glycogen storage enhances the autophagic response to starvation

**Fig 7:**
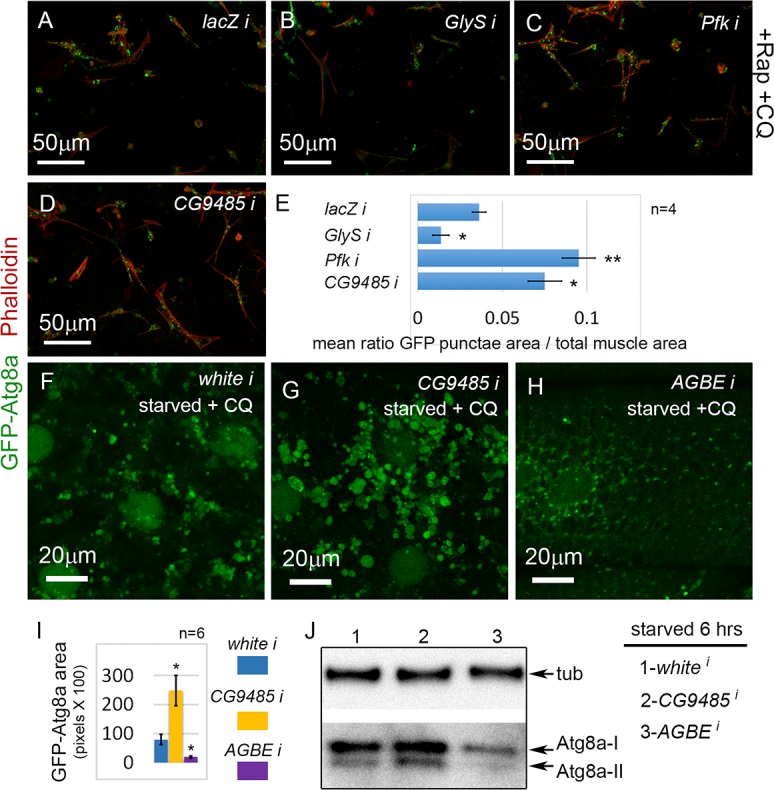
Glycogen metabolism enzymes are autophagy regulators in skeletal muscle. **(A)** Primary cultured muscles with control *lacZ* RNAi, treated for 12 hrs with Rap+CQ. Note the formation of large GFP-Atg8a vesicles throughout the muscles. **(B)** GFP-Atg8a vesicles are reduced in primary cultured muscles with *GlyS* RNAi, treated for 12 hrs with Rap+CQ. Knockdown of *Pfk* (C) or *CG9485* (D) causes increased accumulation of GFP-Atg8a vesicles in primary cultured muscles treated for 12 hrs with Rap+CQ. (**E)** Quantification for A-D of the mean ratio of GFP-Atg8a punctae area to total muscle area per well (n = 4). SEM is indicated with *p<.05; **p<.01. **(F-H)** *Dmef2-Gal4* drives expression of *UAS-GFP-Atg8a* in animals starved on low nutrient food + 1 mg/ml CQ for 6 hrs. **(F)** Control *white* RNAi muscles accumulate large autophagosomes at 1 mg/ml CQ. **(G)** RNAi knockdown of *CG9485* increases autophagosome accumulation in the muscle. **(H)** RNAi knockdown of *AGBE* reduces autophagic vesicle accumulation in the muscle. **(I)** Quantification of the GFP-Atg8a punctae area in muscles from *white*, *CG9485*, and *AGBE* knockdowns.n = 6 larval muscles with SEM indicated and *p<.05. In all panels green = GFP-Atg8a and red = Phalloidin. **(J)** Western blot analysis of endogenous Atg8a from third instar muscle lysate. *Dmef2-Gal4* driven knockdown of *AGBE* but not *CG9485* inhibits Atg8a lipidation, indicating reduced autophagy levels.

Strikingly, several genes, *Pfk (H. sapiens PFKP* and *PFKL*), *CG9485 (H. sapiens AGL)*, and *GlyS (H. sapiens GYS1)*, involved in glycogen biosynthesis were hits in the screen (Fig. 2C). *Glys* encodes glycogen synthase, which we have recently shown is required for glycogen synthesis and autophagosome formation in the *D. melanogaster* musculature [20]. Consistent with this, we found that *Glys* knockdown reduced autophagy in the primary culture screen (Fig. 7B, E; Table 2). In contrast, knockdown of either *Pfk* (Fig. 7C, E), which encodes phosphofructokinase, or *CG9485* (Fig. 7D, E), which encodes glycogen debranching enzyme, caused increased autophagy in the primary muscle assay (Table 1; Table 2). To further analyze the role of glycogen branching in muscle autophagy, we examined the effect of *CG9485* knockdown *in vivo*. We first reduced the CQ feeding treatment from 2.5 to 1mg/ml, so that the enhancement of autophagy would be easier to visualize. Control *white* RNAi larvae starved for 6 hrs and treated with this lower CQ concentration still accumulated autophagosomes (Fig. 7F), but fewer than in larvae treated with 2.5 mg/ml CQ (S3C Fig.). Consistent with the results of the primary culture screen, knockdown of *CG9485* in the larval muscle caused a significant increase in autophagic vesicle accumulation compared to the *white* RNAi control (Fig. 7F, G, I), indicating that this enzyme, which promotes glycogen breakdown, also suppresses autophagy. The effect of *CG9485* knockdown was also examined for starved larvae without the addition of CQ, using the GFP-mCherry-Atg8a reporter, which showed significantly increased punctae formation (S3K Fig.), but no significant block of autophagic flux (S3L Fig.). Thus the increased Atg8a punctae caused by CG9485 knockdown is due to increased autophagy induction.

Next, we tested whether the *D. melanogaster* glycogen branching enzyme (AGBE), which carries out the opposite reaction to CG9485, and hence promotes glycogen storage, would likewise have an autophagy phenotype *in vivo. AGBE* RNAi larvae starved for 6 hrs and treated with 1 mg/ml CQ had dramatically reduced GFP-Atg8a accumulation in their muscles compared to both control and *CG9485* RNAi larvae (Fig. 7F-I). The GFP-Atg8a punctae in the *AGBE* RNAi muscles are much smaller than in control larvae (Fig. 7F, H), suggesting that the effect of knockdown may be on autophagosome growth and/or maturation rather than induction. Consistent with this, we did not see a reduction in punctae number with the GFP-mCherry reporter in starved larvae (S3K Fig.), nor an effect on flux (S3L Fig.). Finally, we performed a Western blot of endogenous Atg8a from *CG9485* and *AGBE* RNAi muscles (Fig. 7J). 6 hrs of starvation caused the appearance of the Atg8a-II form in *CG9485* RNAi and control animals, but was absent in the *AGBE* knockdown, consistent with the immunofluorescence data. We also observed an increase in the levels of both Atg8a-I and Atg8a-II in the CG9485 larvae, but the effect was subtle. Taken together, these analyses suggest that the levels of glycogen storage modulate the autophagic response to starvation in the muscle.

## Discussion

### Primary muscle cells as a tool to screen for autophagy genes

Since the identification of the core autophagy genes in yeast, tissue culture cell lines have been the primary tool to evaluate the role and regulation of autophagy in higher organisms. However, since autophagy is a tissue-specific, context dependent process, stable cell lines can only give a limited view of the autophagic process. Further, as cell lines have adapted to the culture medium, sometimes changing ploidy and activating signaling pathways, they can be a misleading indicator of *in vivo* gene function.

To overcome some of the limitations associated with established tissue culture cell lines, we used primary muscle culture system that we previously showed are amenable to RNAi screening [18,23,24]. We established a robust autophagy assay by treatment with Rap and CQ, and were able to suppress autophagy by simply bathing the cells in dsRNA targeting core autophagy components. The primary muscle screen allows for rapid analysis of multiple dsRNA reagents targeting the same gene in a carefully controlled environment. It also allows for the analysis of genes that may have a deleterious effect on animal viability if disrupted *in vivo*. In comparison, one of the difficulties associated with *in vivo* screening for autophagy genes is that autophagosome formation *in vivo* must be carefully controlled as the age, nutritional state, and stress level of the animals, impact autophagy levels. Further, dissection and processing of muscle samples is time consuming and introduces additional variability.

### Validation of the human autophagy protein-protein interaction network

To identify new autophagy proteins that regulate the autophagy pathway, Behrends et al. used mass spectrometry to generate a network of autophagy protein-protein interactions, representing the unstimulated basal state in tissue culture cells [16]. The resulting 409 high-confidence candidate interaction proteins were organized into an autophagy interaction network. Additional RNAi analysis of 86 genes from the network in U2OS human osteosarcoma cells revealed that many of these genes behaved as bone fide autophagy regulators. However, most components of the set have not been validated for their roles in autophagy, even in cultured cell lines. We therefore generated a set of *D. melanogaster* orthologs comprising 40% of the total human high confidence interacting proteins, tested whether any gave an autophagy phenotype in the specific context of muscle cells, and found that 40% of these had an autophagy phenotype in the muscle. This is much greater than the percentage of hits expected from a random set of genes. For example, in another RNAi screen in *D. melanogaster* primary cells, the number of genes affecting muscle morphology was only 4.3% of 1140 randomly chosen genes [18]. In addition, a full genome screen in *D. melanogaster* primary neurons identified only 1% of genes affecting neuronal morphology [46]. While neither of these are an ideal comparison to the primary culture autophagy screen, their much lower hit rates suggest that the screening set we used was highly enriched for autophagy regulators.

### Glucose and glycogen metabolism as autophagy regulators in the muscle

Consistent with the glycolytic metabolism of larval muscles, we identified several genes involved in glucose and glycogen metabolism as autophagy regulators in the muscle. Knockdown of the enzyme phosphofructokinase (*Pfk*), which is essential for glycolysis, and the glycogen debranching enzyme (*CG9485*), caused increased autophagy in the muscle. In humans, mutations in the glycogen debranching enzyme (AGL) and the muscle isoform of PFK cause or Glycogen Storage Disease type III and Glycogen storage disease type VII, respectively [47]. Both diseases are characterized by the accumulation of excessive glycogen in muscles. Interestingly, a recent report showed that PFK deficiency inhibits the ability of rheumatoid arthritis T-cells to activate autophagy [48]. Like *D. melanogaster* skeletal muscles, T-cells rely primarily on glycolysis [49], suggesting that the link between glycogen metabolism and autophagy might be a general property of highly glycolytic cells. In contrast to PFK and debranching enzyme, knockdown of *AGBE*, the *D. melanogaster* glycogen branching enzyme, dramatically reduced the formation of large GFP-Atg8a vesicles. This enzyme normally promotes the formation of properly branched glycogen particles in the muscle. Thus, genes required for efficient glycogen breakdown, *Pfk* and *CG9485*, inhibit starvation-induced autophagy, and a gene responsible for producing glycogen, *AGBE*, promotes starvation-induced autophagy. These observations extend our previous finding that glycogen is a major cargo of autophagosomes ifn the larval muscle, and that GlyS acts as an important regulator of autophagosome formation [20]. That these other glycogen metabolism enzymes influence autophagy suggests that in addition to the specific role of GlyS, overall glycogen levels in the muscle also help determine autophagy levels. A similar correlation has been recently reported in mammalian cardiac muscle, where increased glycogen content in female hearts was also accompanied by increased autophagy [50]. Altogether, our observation that genetic manipulation of glycogen storage impacts muscle autophagy levels has important implications for both skeletal and cardiac myopathies and glycogen storage diseases that are associated with aberrant glycogen autophagy.

### Proteasome subunits as autophagy regulators in our screen

Given that proteasome inhibitors have been previously shown to activate autophagy [35], it was expected that we might identify proteasome subunits as autophagy regulators in our screen. Unexpectedly however, we found that knockdown of proteasome components, *Pros26.4 (H. sapiens PSMC1)*, *Rpt1 (H. sapiens PSMC2)*, and *Rpn1 (H. sapiens PSMD2)*, inhibited autophagosome formation. Knockdown of other proteasome subunits gave the opposite phenotype. The variation in the direction of proteasome phenotypes is likely due to the level of proteasome dysfunction rather than to the specific roles of the proteasome subunits, since knockdown of subunits in the larval muscle consistently inhibited autophagosome formation. Our data indicates that the sustained, high level of proteasome inhibition achieved by subunit knockdown is incompatible with autophagy. However, short-term proteasome inhibition via overexpression of temperature sensitive proteasome alleles did induce an autophagic response, consistent with previous reports for both *D. melanogaster* muscle and diverse mammalian tissues [39,40,51]. Surprisingly, we found that both ubiquitin aggregates and GFP-Atg8a punctae were reduced after about a day of proteasome inhibition *in vivo*, accompanied by progressive locomotor dysfunction. Tyramine treatment inhibited locomotion and likewise reduced the formation of aggregates and autophagosomes. We propose that proteasome inhibition induces a burst of aggregate formation and autophagy in the muscle, but diminished muscle activity subsequently suppresses both. Strenuous physical activity has been linked to the production of reactive oxygen species and mechanical damage of mitochondria in muscle [52] and to autophagy in the *D. melanogaster* flight muscle [53]. Our data suggests that larval muscle contraction may drive the accumulation of aggregates and increased autophagy that manifest during proteasome inhibition.

### Roles of Rab GTPases and a Rab effector during autophagy

It has recently become clear that Rab GTPases play critical roles in membrane trafficking events during autophagy. This large family of small monomeric GTPases localize to different membranes in the cell, where they recruit effector proteins that carry out membrane transport functions. We identified the Rab effector CG41099 (ANKFY1) as an important regulator of autophagosome formation in the muscle system (Fig. 5E, N). ANKFY1 was previously identified as a Rab5 effector that localizes to early endosomes and stimulates their fusion activity [44]. Depletion of ANKFY1 in human cell culture experiments led to increased autophagosomal number without blocking flux [16], however, we observed that knockdown of *CG41099* inhibited the formation of enlarged CQ-induced vesicles and increased the amount of autolysosomes compared to autophagosomes. This contradiction could reflect real biological differences between the two species and cell types or reflect differences in the assays. The effect of *CG41099* knockdown on flux is consistent with a previous report that ANKFY1 can localize to the endosomal and lysosomal membranes [44].

Rab GTPase-activating proteins (Rab GAPs) negatively regulate the activity of Rab GTPases by catalyzing the conversion of GTP to GDP [54]. Interestingly, several Rab GAPs are recruited to autophagosomes by direct interactions with ATG8 homologs, where they regulate autophagosome maturation and fusion events [55]. For example, the Rab GAP TBC1D5 mediates autophagosome maturation and has been proposed to act as a molecular switch between endosome and autophagosome biogenesis [55]. Through our screen and *in vivo* follow-up, we identified the Rab GTPase-activating proteins CG7112 (*H. sapiens* RABGAP1 and RABGAP1L), CG31935 (*H. sapiens* RAB3GAP1), and Rab3-GAP (*H. Sapiens* RAB3GAP2) as regulators of autophagosome formation in muscles. RABGAP1 and RABGAP1L have previously been shown to act on Rab6 and Rab22A, 34, 39B, respectively [56]. RABGAP1, RABGAP1L, and RAB3GAP1 have each been shown to interact with ATG8 homologs [16,55], but to our knowledge, none have been shown to have an *in vivo* function in autophagy prior to this study. RAB3GAP1 and RAB3GAP2 are the catalytic and non-catalytic subunits of the Rab3GAP complex, which acts on Rab3 isoforms [57,58]. Mutations in these genes, which have heightened expression in the brain, cause Micro syndrome and Martsolf syndrome, related rare disorders characterized by microcephaly, microphthalmia, cataracts and intellectual disability [59]. *D. melanogaster CG31935* and *Rab3-GAP* mutant neurons have a defect in neurotransmitter release that is suppressed in *Rab3* mutants [60]. Our results indicate that the function of these proteins in muscle autophagy is not mediated through Rab3, since neither *Rab3* mutants nor expression of a constitutively active form of Rab3 had an effect on autophagosome formation. Finally, *Rab3* expression in *D. melanogaster* is very low outside of the central nervous system.

Further research will be needed to establish how CG7112, CG31935 and Rab3GAP influence membrane trafficking and autophagosome formation, particularly, which Rab proteins mediate their effect in muscle. Intriguingly, we found that expression of a constitutive active form of Rab39 caused an autophagy phenotype similar to *CG7112*, *CG31935* and *Rab3-GAP* knockdown, suggesting that Rab39 may function downstream of one of these Rab GAPs during autophagy regulation. The suppression of autophagy that we observed by overexpression of constitutively active Rab39 is consistent with a previous report that mammalian Rab39a negatively regulates LPS-induced autophagy in macrophages[61]. Our results also highlight that the role of the human *RAB3GAP* genes in the etiology of Micro and Martsolf syndromes might involve defects in autophagy, in addition to other vesicle trafficking pathways.

## Materials and Methods

### Fly stocks

*yw; Dmef2-Gal4* [62]; *Dmef2-Gal4* was recombined with *UAS-GFP-Atg8a* [63]; *UAS-GFP-mCherry-Atg8a* [36]; *UAS-GFP-Lamp1* [64]; *UAS-DTS5* [42]; *Rab3* ^*rup*^ [65]; *UAS-CFP-Rab3-GAP* [60]; *UAS-YFP.Rab3.Q80L* [66]; *UAS-YFP.Rab5.Q88L* [66]; *UAS-YFP.Rab39.Q69L* [66]. The following UAS-RNAi lines were obtained from the DRSC/TRiP DRSC/TRiP at Harvard Medical School (http://www.flyrnai.org/TRiP-HOME.html): *white* (HMS0004); *Rpn1* (HMS01337); *Prosalpha5* (HMS00095); *Prosbeta5* (HMS00119); *Prosbeta7* (HMS00122); *CG9588* (HM05013); *Mov34* (JF01140); *Rpn9* (HMS01007); *Rpn12* (HMS01032); *CG41099* (HMS01228); *CG7112* (HMS01132); *Rab3-GAP* (JF01601); *CG9485* (HMS01321); *AGBE* (HMS02027). The RNAi line targeting *CG31935* (NIG31935R-1) was obtained from the NIG Japan. Unless otherwise noted, flies were reared at 25°C.

### Generation of *UAS-myc-Atg8a* transgenic flies

Atg8a cDNA LD05816 was cloned into the Gateway entry vector according to the pENTR Directional TOPO Cloning system (Invitrogen), then cloned into pTMW = N-terminal 6xMyc tag under the control of the UASt promoter. This destination vector was created at the *Drosophila* Gateway Collection, and obtained from the *Drosophila* Genomics Resource Center. Integration into the genome was performed using standard methods.

### Feeding protocol, and drug treatment

All fly crosses and larvae were maintained in vials containing ‘standard’ food composed of 16.5 g/L yeast, 9.5 g/L soy flour, 71 g/L cornmeal, 5.5 g/L agar, 5.5 g/L malt, 7.5% corn syrup, 0.4% propionic acid, and 1.3% Tegosept. For starvation induced autophagy experiments 3rd instar larvae were individually selected and no more than 20 per experiment were transferred to ‘low nutrient food’ composed of 0.3X standard food as described previously [20]. For each genotype, at least 6 larvae from at least two independent vials were analyzed. For drug treatments, chloroquine diphosphate salt (Sigma) was added to the food at 2.5 mg/ml or 1.0 mg/mg as indicated in the text, and tyramine (Sigma) was added at 50 mg/ml or 100 mg/ml 18 hrs prior to dissection.

### Temperature sensitive alleles

Prior to experiments larvae were reared at 22°C. Vials were transferred to the restrictive temperature of 29°C to induce proteasome inhibition and maintained at this temperature for the duration of the experiment. Transfer to starvation food +/- CQ was done 6hrs prior to dissection.

### Larval locomotor assays

Assays were based on previously published methods [20,67]. For the crawling assay, larvae were positioned at one end of a furrow on the surface of a sylgard plate, with a yeast ball at the far end. The time to crawl 3cm was measured 3 times, with the final successful trial used as data for analysis. Trials in which the larva crawled over the edge of the lane were considered unsuccessful, and the larvae were reset at the starting point.

### Larval muscle immunostaining and antibodies

For whole-mount immunostaining of fly tissues, 3rd instar larval body wall muscles were dissected according to [68] and fixed for 15 min in PBS with 4% formaldehyde. After washing in PBT (1X PBS + 0.1% Triton X-100), samples were incubated overnight with the following antibodies (in PBT): mouse anti-Poly-Ubiquitin, 1:300 (FK2; Enzo life sciences), Rabbit anti-myc-tag, 1:200 (71D10; Cell Signaling). After incubation with primary antibodies, the samples were washed in PBT and incubated with Alexa-conjugated secondary antibodies (Molecular Probes, 1:1000) and/or Alexa 635-conjugated phalloidin (1:1000) to visualize F-actin. Nuclei were visualized by DAPI staining (1μg/ml). Samples were washed in PBT and mounted in 1:1 glycerol/PBS and images were acquired with a Leica SP2 laser scanning confocal microscope.

### Larval muscle western blots

Prior to dissection larvae were fed on standard food, then 10–12 larvae of each genotype were transferred to new vials containing either standard food or low-nutrient food for 6 hrs. Third instar larvae were dissected to obtain a carcass with only muscles remaining attached. Dissections were collected in lysis buffer (120mM NaCl, 50mM Tris-HCl, 1% Nonidet P-40, 1× protease and phosphatase inhibitor cocktail [Thermo Scientific]). After homogenization, debris was removed by centrifuging once at 1,200 × g for 5 min and once at 13,000 × g for 5 min. Western blot was performed using standard protocols. Antibodies used are: rabbit anti-dAtg8 [37] (1:1000, gift from Katja Köhler) and mouse anti-Tubulin (1:5,000 Sigma-Aldrich T6199).

### Larval muscle image analysis

For quantification of autophagy area, z-stacks at 40X of single larval longitudinal muscles were obtained by confocal microscopy with consistent laser power settings. ImageJ was used to produce a maximum intensity projection of the stack [69]. Threshold of the GFP-Atg8a channel was produced with the Robust Automatic Threshold Selection (RATS) Plugin with the noise threshold = 20, lambda factor = 3, and min leaf size = 100. This allowed for the elimination of background signal not beloning to any autophagosomal structure, and the area of the remaining signal was calculated to determine the GFP-Atg8a punctae area per muscle. At lease five ventral longitudinal muscles from individual animals were analyzed for each genotype. For quantification of autophagic vesicle and ubiquitin puncta number single confocal sections were obtained at 40X at the level of the nucleus with consistent laser power. ImageJ was used to threshold the red and green channels (with RATS as above) and count the GFP-Atg8a and ubiquitin dots in single ventral longitudinal muscles of 8 or more individual animals. The Manders’ Overlap Coefficient (MOC) measurement of red and green channel colocalization was calculated using a previously described ImageJ Plugin [32]. For all image analysis mean values were plotted, with error bars indicating the standard error of the mean. p-values were calculated by Student’s t-test.

### Identification of orthologs

*D. melanogaster* orthologs of the 409 high-confidence candidate interaction proteins previously identified by mass-spec [16] were identified by comparing the fruit fly and human genomes using the OrthoMCL algorithm [70]. Human and fly protein sequences were extracted from UniProt (r.2010_04) and FlyBase (r.5.24), respectively. The resulting protein sequence database was used to run all-against-all protein sequence comparisons with BLASTP and subsequent MCL clustering of a sequence similarity matrix, as described in [70]. Based on empirical studies, an inflation value of 1.5 was selected to control cluster tightness during MCL clustering. Also, an e-value of 1e-10 and 65% sequence similarity threshold were chosen to obtain orthologous and co-orthologous protein pairs.

### Preparation of dsRNAs

Gene-specific dsRNAs were prepared as described previously [71]. Briefly, amplicons (∼200–500 bp) were amplified by PCR from genomic DNA, using synthesized oligos with an attached T7 sequence. dsRNAs from the PCR templates were transcribed with the T7 Megascript kit (Ambion), and product size was confirmed by gel electrophoresis. Following purification with Millipore Multiscreen PCR plates (#MANU03050), dsRNAs were quantified by measurement of the OD260 (Nano-drop 8000, Fisher Scientific), then stored at-20°C until use.

### Primary cell culture RNAi experiments

Embryonic primary cell cultures were isolated from gastrulating *Dmef2-Gal4*, *UAS-GFP-Atg8a* embryos as described previously [18,23,46,71], and seeded in 384-well plates at 4 X 10^4^ cells (10ml volume) per well. Each well contained 5ul dsRNA in water (0.25ug dsRNA) targeting a gene from the autophagy network or control dsRNAs targeting *lacZ* (negative control), *Atg18* (positive control) or *thread* (RNAi knockdown control). Following 20 hours incubation in serum-free M3 medium at 18°C, 30μl of serum-containing medium was added to each well for a final fetal calf serum concentration of 10%. Cells were then cultured for an additional 3 days at 25°C before addition of rapamycin (200 nM) and chloroquine (200uM). Cells were incubated for 12 hrs at 25°C before fixation for 2 hr in 2% formaldehyde. Cells were washed and stained overnight at 4°C with phalloidin Alexa Fluor 635 (Molecular Probes; 1:2000), and DAPI (Sigma, 1:5000), then washed again. All washes were performed in PBT, except for a final rinse in PBS prior to image analysis. This experiment was repeated to give 4 biological replicates of each amplicon.

### Primary cell image analysis

Acquisition of high quality images of the primary cell culture was performed with the Evotec Opera microscope at the DRSC (www.flyrnai.org/). Using a 20X water immersion lens, 24 microscope fields were obtained per well for both GFP-Atg8a and phalloidin stains. Images were analyzed with MetaXpress High Content Image Acquisition & Analysis Software (Molecular Devices). Muscles were identified from the mixed population of cells by positive phalloidin staining as described previously [71]. Then, GFP-Atg8a vesicles were identified within these muscles using the MetaXpress granularity application module. Total GFP-Atg8a area was divided by total muscle area for each field. These values were then combined to give a measure of the total area of autophagosomes per muscle cell area per well. We used the MATLAB-based open-access software tool, GUItars, for subsequent calculation of SSMD scores of the RNAi screening data [72]. Strictly standardized mean difference (SSMD) of log-transformed data is the mean of log fold change divided by the standard deviation of log fold change between a dsRNA and a control reference [34]. The GUItars software uses the uniformly minimal variance unbiased estimate (UMVUE) of SSMD in the sample scoring [72,73]. To account for plate variability, the software first calculates the difference between the measured autophagosome area of given well and the median of a negative control (*lacZ*) in each plate and then calculates the SSMD. A gene was considered a regulator of autophagosome formation if two or more amplicons targeting that gene met the threshold (±0.5 SSMD). Other genes were considered for analysis if a single amplicon targeting the gene met the threshold (±1.0 SSMD) and none of the other amplicons targeting that gene met the threshold (±0.5 SSMD) in the opposite direction.

## Supporting Information

S1 Table List of all amplicons screened in primary cultured muscles. *D. melanogaster* genes (column A) are matched to their human orthologs (column B). SSMD scores (column C) are listed for each amplicon (column D). The data is organized into two tabs. The first shows all of the screened amplicons that were derived from the human autophagy interaction network [16]. The second tab lists additional genes and amplicons included on the screening plates.
(XLSX)

S1 Fig Imaging of autophagy in primary cultured muscles. **(A)** Primary cultures from *D. melanogaster* embryos expressing GFP-Atg8a under the control of *Dmef2-Gal4*, stained with Phalloidin (actin) in red. Addition of chloroquine (CQ) and rapamycin (Rap), results in the accumulation of large GFP-Atg8a labeled vesicles. **(B)** Muscles are segmented and counted using the MetaXpress neurite outgrowth module. **(C)** The number and area of autophagosomes are counted using the MetaXpress granularity module. **(D)** Overlay of (B) and (C) eliminates any GFP signal outside of the muscles, allowing for calculation of autophagosome area and number per muscle number and area.
(TIF)

S2 Fig SSMD plot of a 384-well experimental plate. Scatter plot of SSMD scores for dsRNA amplicons from 3 biological replicates of one 384 well plate from the autophagy set. Blue dots are the various dsRNAs tested, with positive scores indicating increased autophagosome formation, and negative scores indicating reduced autophagosome formation. Orange squares are the negative *lacZ* dsRNA control wells. Note that these always score between-0.5 and 0.5. Green triangles represent *Atg18* dsRNA positive control wells. As expected, these wells always give a score below-1.5, indicating a strong suppression of autophagosome formation.
(TIF)

S3 Fig In vivo autophagy assays. **(A-D)** *Dmef2-Gal4* drives *UAS-GFP-Atg8a* specifically in the larval muscles. **(A)** In fed larval muscle, Atg8a is distributed throughout the cytoplasm and nucleus. **(B)** In starved larval muscle, Atg8a localizes to small punctae. **(C)** In starved larval muscle treated with CQ, Atg8a localizes to enlarged autophagosomes. **(D)** Knockdown of *Atg1* inhibits the formation of autophagosomes in starved + CQ muscles. **(E)** Quantification of phenotypes from A-D. SEM is indicated, with n = 8 ventral longitudinal muscles from individual animals and ***p<.001. **(F-I)** *Dmef2-Gal4* drives *UAS-GFP-mCherry-Atg8a* in the larval muscles. **(F)** In fed animals GFP and mCherry colocalize throughout the cytoplasm and nuclei of the muscle. **(G)** 6 hrs starvation induces punctae labeled primarily by both GFP and mCherry, indicating that acidification of vesicles has not yet occurred. **(H)** After 8 hrs starvation, some punctae have completely lost GFP labeling. **(I)** After 10 hrs starvation, most punctae are labeled with mCherry, but not GFP. **(J)** Quantification of the ratio of mCherry/GFP-mCherry punctae from F-I. SEM is indicated, with n = 6 ventral longitudinal muscles from individual animals and **p<.01; ***p<.001. **(K-L)** Quantification of phenotypes for *Dmef2-Gal4* driven expression of *DTS* mutant or knockdown of screen hits. Starvation 6hrs +/- CQ is indicated. **(K)** Total GFP-mCherry-Atg8a punctae. **(L)** Ratio of mCherry/GFP-mCherry punctae. SEM is indicated, with n = 6 ventral longitudinal muscles from individual animals. P-values for knockdown experiments are relative to *white* RNAi controls with *p<.05; **p<.01; ***p<.001.
(TIF)

S4 Fig Knockdown of proteasome subunits *in vivo* inhibits autophagosome formation. *Dmef2-Gal4* drives expression of *UAS-RNAi* constructs targeting *white* and selected components of both 19S and 20S subunits of the proteasome, *Prosbeta5 (PSMB5)*, *Prosbeta7 (PSMB4)*, *Prosalpha5 (PSMA5)*, *CG9588 (PSMD9)*, *Mov34 (PSMD7)*, *Rpn9 (PSMD13)*, *Rpn12 (PSMD8)*. For each genotype animals were fed on standard food or starved and treated with CQ to induce autophagy. Proteasome subunit knockdown caused different degrees of ubiquitin aggregate accumulation, but autophagosome formation was always less than in *white RNAi* control muscles. In all panels green = GFP-Atg8a, red = anti-ubiquitin, and blue = DAPI.
(TIF)

S5 Fig *Rpn1* knockdown reduces GFP-Lamp1 vesicles. *Dmef2-Gal4* drives expression of *UAS-GFP-Lamp1* and *UAS-RNAi* constructs targeting *white* and *Rpn1*. (A) In fed *white* RNAi larvae, GFP-Lamp1 localizes to the muscle cytoplasm and to a small number of punctae. (B) In *white* RNAi larvae starvation for 6hrs triggers increased GFP-Lamp1 localization to small punctae. (C) *Rpn1* RNAi significantly reduces the number of GFP-Lamp1 labeled punctae in response to starvation. (D) 6hrs starvation + CQ treatment induces the number of GFP-Lamp1 punctae in *white* RNAi larvae. (E) *Rpn1* RNAi significantly reduces the number of GFP-Lamp1 punctae induced by 6hrs starvation + CQ treatment. (F) Quantification of mean number of GFP-Lamp1 punctae from A-F. SEM is indicated, with n = 10 muscles and p-values for *Rpn1* RNAi shown relative to *white* RNAi controls (***p<.001). In panels A-E, green = GFP-Lamp1 and blue = DAPI.
(TIF)
