# Supplementary figures and images for: Regulators of Autophagosome Formation in Drosophila Muscles
Source: PLoS Genet. 2015 Feb 18;11(2):e1005006. doi: 10.1371/journal.pgen.1005006 (PMC4334200; doi:10.1371/journal.pgen.1005006)

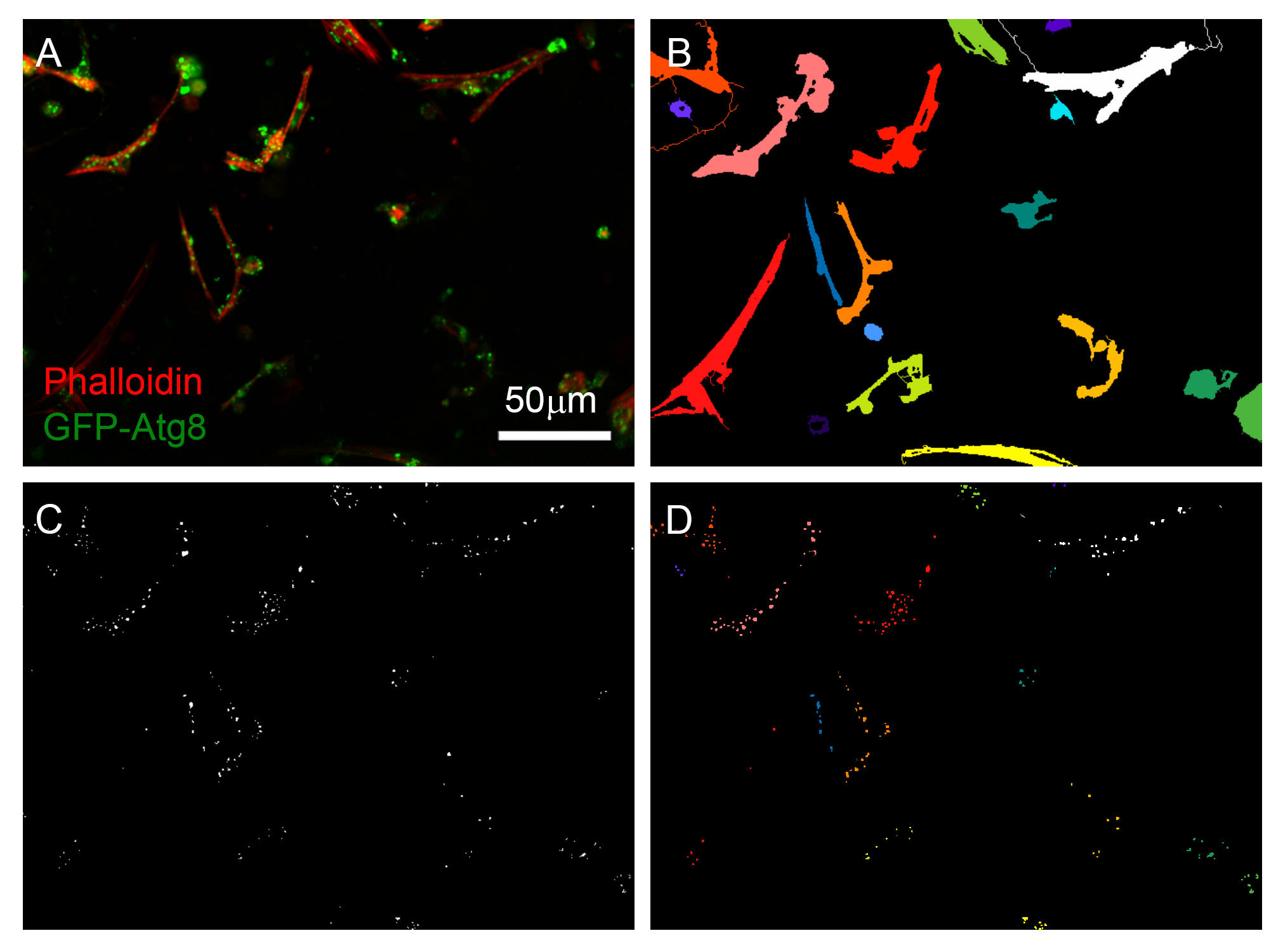

Supplement: S1 Fig — (A) Primary cultures from D. melanogaster embryos expressing GFP-Atg8a under the control of Dmef2-Gal4, stained with Phalloidin (actin) in red. Addition of chloroquine (CQ) and rapamycin (Rap), results in the accumulation of large GFP-Atg8a labeled vesicles. (B) Muscles are segmented and counted using the MetaXpress neurite outgrowth module. (C) The number and area of autophagosomes are counted using the MetaXpress granularity module. (D) Overlay of (B) and (C) eliminates any GFP signal outside of the muscles, allowing for calculation of autophagosome area and number per muscle number and area. (TIF) [file pgen.1005006.s002.tif]

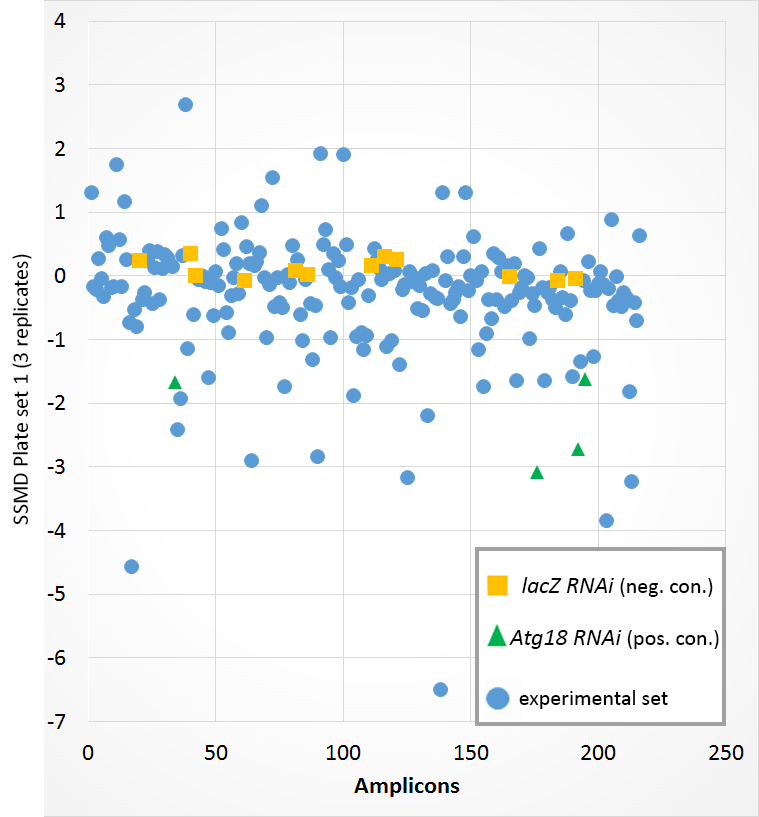

Supplement: S2 Fig — Scatter plot of SSMD scores for dsRNA amplicons from 3 biological replicates of one 384 well plate from the autophagy set. Blue dots are the various dsRNAs tested, with positive scores indicating increased autophagosome formation, and negative scores indicating reduced autophagosome formation. Orange squares are the negative lacZ dsRNA control wells. Note that these always score between-0.5 and 0.5. Green triangles represent Atg18 dsRNA positive control wells. As expected, these wells always give a score below-1.5, indicating a strong suppression of autophagosome formation. (TIF) [file pgen.1005006.s003.tif]

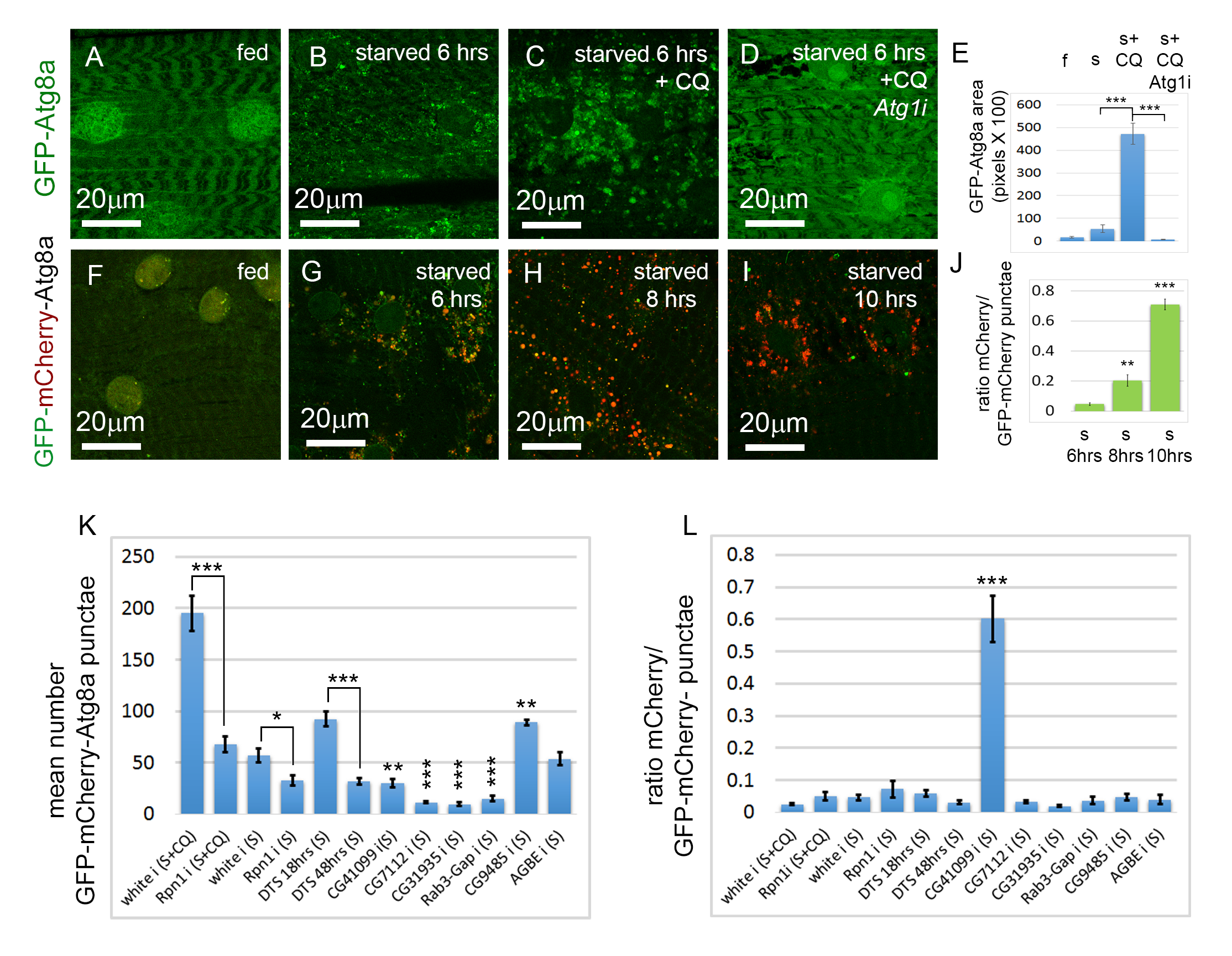

Supplement: S3 Fig — (A-D) Dmef2-Gal4 drives UAS-GFP-Atg8a specifically in the larval muscles. (A) In fed larval muscle, Atg8a is distributed throughout the cytoplasm and nucleus. (B) In starved larval muscle, Atg8a localizes to small punctae. (C) In starved larval muscle treated with CQ, Atg8a localizes to enlarged autophagosomes. (D) Knockdown of Atg1 inhibits the formation of autophagosomes in starved + CQ muscles. (E) Quantification of phenotypes from A-D. SEM is indicated, with n = 8 ventral longitudinal muscles from individual animals and ***p<.001. (F-I) Dmef2-Gal4 drives UAS-GFP-mCherry-Atg8a in the larval muscles. (F) In fed animals GFP and mCherry colocalize throughout the cytoplasm and nuclei of the muscle. (G) 6 hrs starvation induces punctae labeled primarily by both GFP and mCherry, indicating that acidification of vesicles has not yet occurred. (H) After 8 hrs starvation, some punctae have completely lost GFP labeling. (I) After 10 hrs starvation, most punctae are labeled with mCherry, but not GFP. (J) Quantification of the ratio of mCherry/GFP-mCherry punctae from F-I. SEM is indicated, with n = 6 ventral longitudinal muscles from individual animals and **p<.01; ***p<.001. (K-L) Quantification of phenotypes for Dmef2-Gal4 driven expression of DTS mutant or knockdown of screen hits. Starvation 6hrs +/- CQ is indicated. (K) Total GFP-mCherry-Atg8a punctae. (L) Ratio of mCherry/GFP-mCherry punctae. SEM is indicated, with n = 6 ventral longitudinal muscles from individual animals. P-values for knockdown experiments are relative to white RNAi controls with *p<.05; **p<.01; ***p<.001. (TIF) [file pgen.1005006.s004.tif]

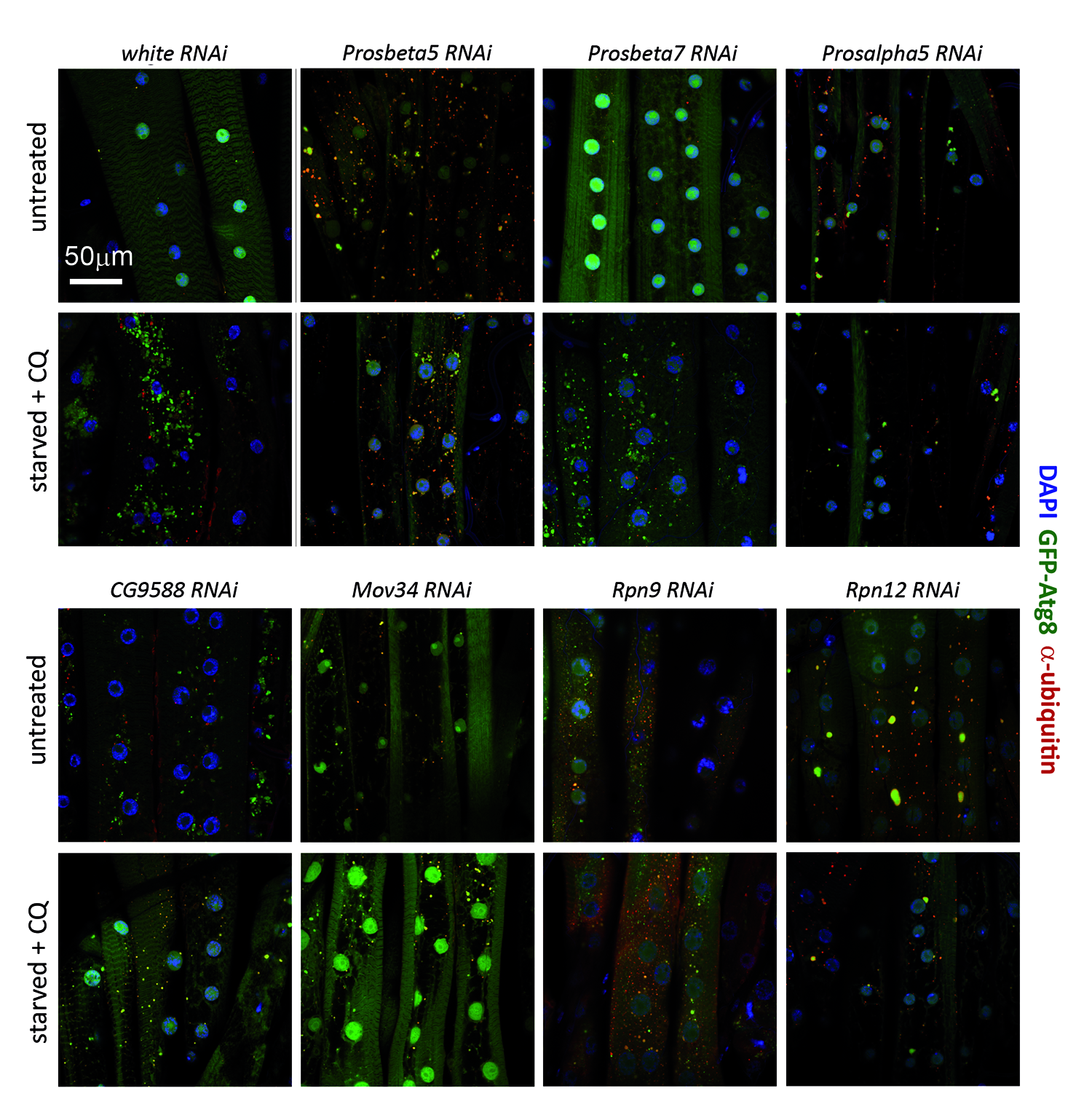

Supplement: S4 Fig — Dmef2-Gal4 drives expression of UAS-RNAi constructs targeting white and selected components of both 19S and 20S subunits of the proteasome, Prosbeta5 (PSMB5), Prosbeta7 (PSMB4), Prosalpha5 (PSMA5), CG9588 (PSMD9), Mov34 (PSMD7), Rpn9 (PSMD13), Rpn12 (PSMD8). For each genotype animals were fed on standard food or starved and treated with CQ to induce autophagy. Proteasome subunit knockdown caused different degrees of ubiquitin aggregate accumulation, but autophagosome formation was always less than in white RNAi control muscles. In all panels green = GFP-Atg8a, red = anti-ubiquitin, and blue = DAPI. (TIF) [file pgen.1005006.s005.tif]

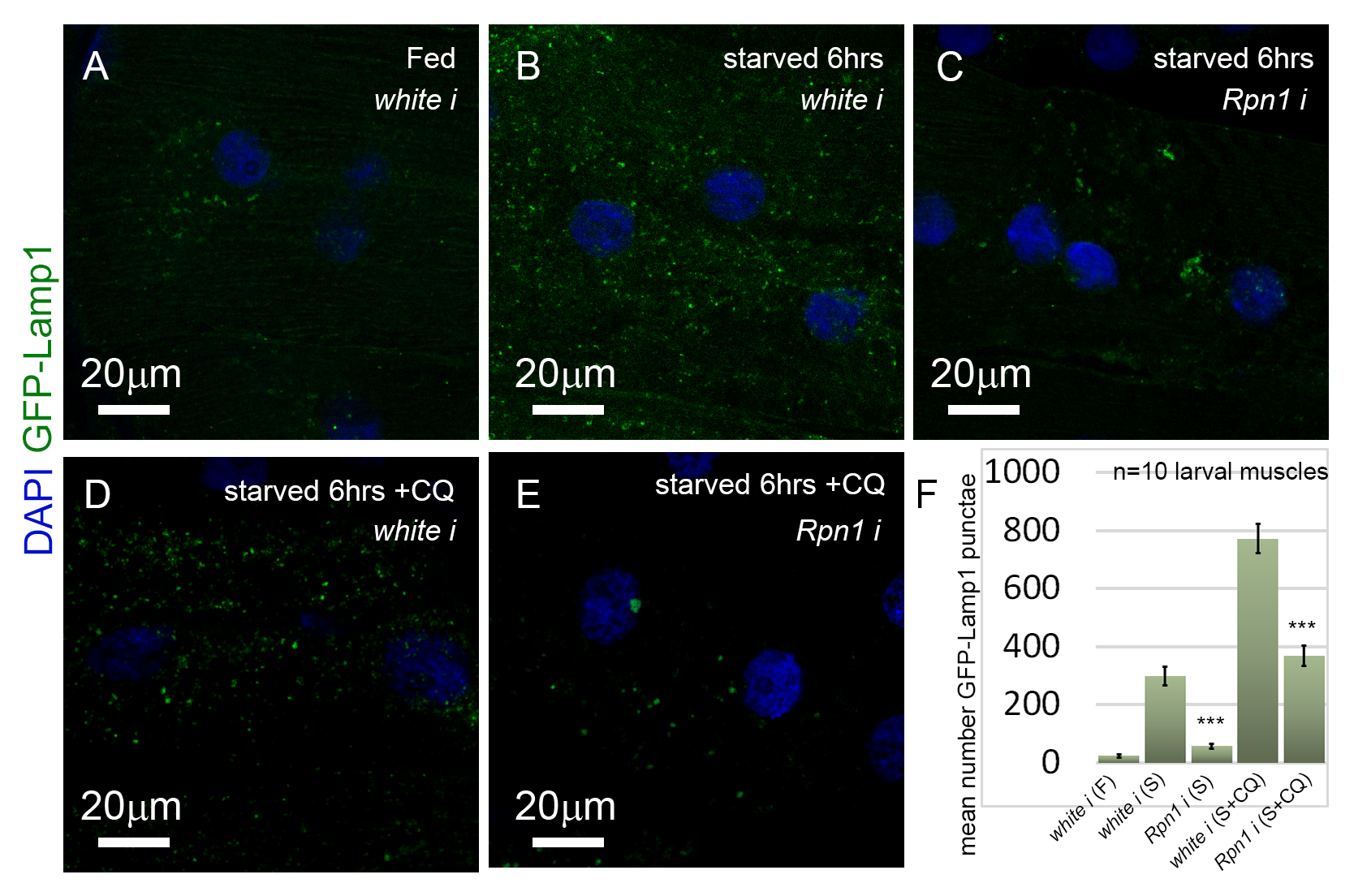

Supplement: S5 Fig — Dmef2-Gal4 drives expression of UAS-GFP-Lamp1 and UAS-RNAi constructs targeting white and Rpn1. (A) In fed white RNAi larvae, GFP-Lamp1 localizes to the muscle cytoplasm and to a small number of punctae. (B) In white RNAi larvae starvation for 6hrs triggers increased GFP-Lamp1 localization to small punctae. (C) Rpn1 RNAi significantly reduces the number of GFP-Lamp1 labeled punctae in response to starvation. (D) 6hrs starvation + CQ treatment induces the number of GFP-Lamp1 punctae in white RNAi larvae. (E) Rpn1 RNAi significantly reduces the number of GFP-Lamp1 punctae induced by 6hrs starvation + CQ treatment. (F) Quantification of mean number of GFP-Lamp1 punctae from A-F. SEM is indicated, with n = 10 muscles and p-values for Rpn1 RNAi shown relative to white RNAi controls (***p<.001). In panels A-E, green = GFP-Lamp1 and blue = DAPI. (TIF) [file pgen.1005006.s006.tif]
